# The chronology of Gezer from the end of the late bronze age to iron age II: A meeting point for radiocarbon, archaeology egyptology and the Bible

**DOI:** 10.1371/journal.pone.0293119

**Published:** 2023-11-15

**Authors:** Lyndelle C. Webster, Samuel R. Wolff, Steven M. Ortiz, Marcella Barbosa, Cameron Coyle, Gary P. Arbino, Michael W. Dee, Quan Hua, Geraldine E. Jacobsen

**Affiliations:** 1 Austrian Archaeological Institute, Austrian Academy of Sciences, Vienna, Austria; 2 W. F. Albright Institute for Archaeological Research, Jerusalem, Israel; 3 Lanier Center for Archaeology, Lipscomb University, Nashville, TN, United States of America; 4 Gateway Seminary, Ontario, CA, United States of America; 5 Centre for Isotope Research (CIO), University of Groningen, Groningen, Netherlands; 6 Australian Nuclear Science and Technology Organisation, Sydney, Australia; 7 School of Social Science, The University of Queensland, Brisbane, Australia; New York State Museum, UNITED STATES

## Abstract

The ancient southern Levantine city of Gezer is well-known from Egyptian, Biblical and Assyrian sources, associated with power struggles, conquests, and intriguing tales involving figures such as Milkilu and Amenhotep III, Merneptah, the Philistines, Solomon and his unidentified pharaonic father-in-law, and Shishak / Sheshonq I. Since the identity of Gezer with “Tell Jezer” is quite literally ‘set in stone’ by some dozen boundary inscriptions, along with impressive Bronze and Iron Age remains, research at this site provides a unique opportunity to compare text and archaeology, as well as bring to light the undocumented everyday lives of the city’s inhabitants. In this endeavour, independent scientific dating is crucial for anchoring the remains chronologically. This paper presents the first substantial radiocarbon dataset and Bayesian chronological analysis for Gezer spanning the last part of the Late Bronze Age (LBA; LB IIB) through Iron Age II. The dataset derives from an essentially continuous stratigraphic sequence exposed in recent years by the Tandy expedition along the central-southern edge of the site. The results allow us for the first time to independently determine the site chronology, test the viability (from a chronological perspective) of proposed historical correlations, and contribute to debates on Philistine and Iron Age chronology.

## Introduction

**Fig 1:**
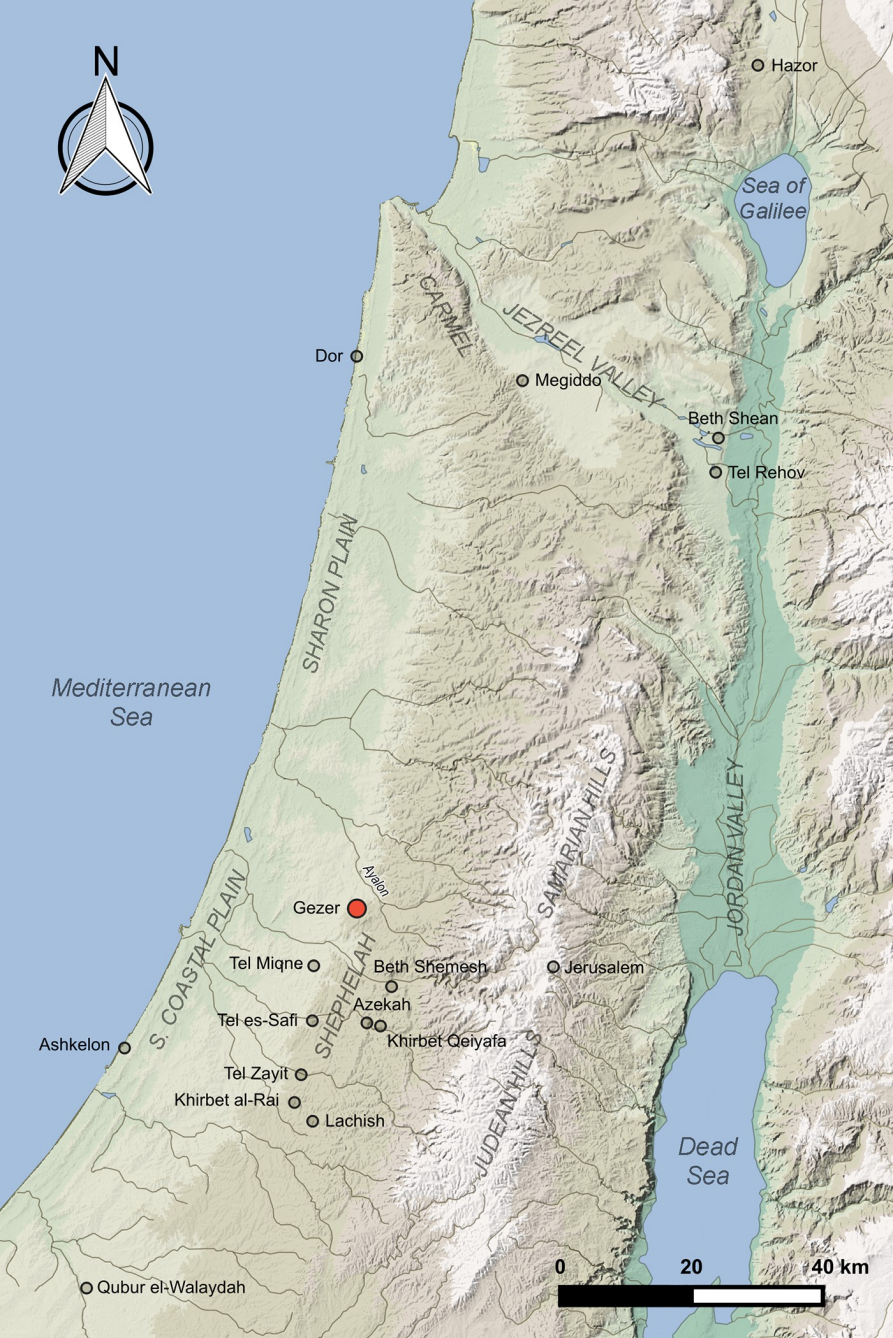
Location of Gezer and sites mentioned in the text.

For scholars seeking to reconstruct the Bronze and Iron Age history of the southern Levant and explore the interplay between text and archaeological evidence, Gezer is among the most intriguing sites. This ancient city is mentioned in many textual sources and its strategic importance is amply attested by the attention it received from foreign rulers. Gezer is securely identified with Tell Jezer (ITM/NIG grid ref. 192513, 640728), which at ca. 12 ha is among the largest mounds in the southern Levant and easily the largest in the Shephelah (foothill) region of south-central Israel. Gezer is located at a key crossroad between the Via Maris coastal road and the road leading inland to the highlands and beyond to Transjordan (Fig 1). Sitting atop a promontory at 225 m elevation, it commands an impressive, almost 360-degree view of the surrounding terrain: across the southern coastal and Sharon plains and eastward to the Judean hills. The inhabitants had access to wells and springs, and to fertile fields in the Ayalon and adjacent valleys.

### Gezer in historical and biblical sources

Gezer is mentioned in Egyptian, Assyrian and biblical texts–sources that carry varying weight for reconstructing history. The Egyptian and Assyrian texts are contemporary with the events they describe and thus generally accepted as describing real events (notwithstanding political biases of the authors). The biblical texts were written centuries later and thus the historical realities behind them are less clear and more strongly debated.

Gezer was well-known to the Egyptians since at least the 18^th^ Dynasty. During the 15^th^ century BC, this town appears in the topographic list of Thutmose III, and Thutmose IV claims to have captured Hurrians nearby [1]. The chronology of these rulers is supported by radiocarbon dating, with accession years estimated in the range 1518–1501 BC and 1434–1420 BC respectively (95.4% probability) [2–4]. By the 14^th^ century BC, Gezer was one of the dominant city-states in central-southern Canaan, its rulers featuring prominently in the Amarna correspondence [5, 6]. Towards the end of the Late Bronze Age, Merneptah (accession 1241–1219 BC, 95.4%) launched a campaign into southern Canaan, evidently to quell a rebellion that broke out at the end of Ramesses II’ long reign [7, 8]. Gezer is one of few sites singled out as having been captured. In his stele we read:

“Carried off is Canaan with every evil, Brought away is Ascalon, taken is **Gezer**, Yenoam is reduced to non-existence; Israel is laid waste having no seed.” [9]

The historicity of Merneptah’s southern Levantine campaign and his attack on Gezer (dated to his 5^th^ year) enjoys wide acceptance [10]. Further evidence comes from the Amada Stela, where the king proudly titles himself as the “subduer of Gezer” [9] and the event may even be depicted in a battle relief at Karnak [11].

Whether the biblical text preserves memories of Gezer as a prominent Canaanite centre is debated [12, 13]. The Bible is a major source for the Iron Age southern Levant, and though written down centuries later, most scholars consider that it reflects some early realities. The overall evidence suggests that by the early Iron Age, Gezer lay at the border between emerging coastal and highland polities and was a frontier for conflict: “there arose a war with the Philistines at Gezer” (1 Chr. 20:4; see also 2 Sam. 5:25 and 1 Chr. 14:16).

During the timeframe of the debated ‘United Monarchy’, Gezer appears in several intriguing texts. 1 Kings 9:15–17 mentions Gezer’s capture, burning and presentation as a wedding gift by Solomon’s father-in-law–an unnamed Egyptian king; it then claims that Solomon proceeded to build up Gezer, along with Megiddo, Hazor and other towns. Scholarly views vary regarding the composition and redaction of this text and the mix of early and/or later realities reflected (e.g. [14–16]). For those who would see a historical capture of Gezer during the 10^th^ century BC, Siamun of the 21^st^ Dynasty has most often been suggested as the unnamed king [17–20].

Sheshonq I, the Libyan founder of the 22^nd^ Egyptian dynasty (accession 988–945 BC, 95.4%), left a toponym list and triumphal relief at Karnak that includes many southern Levantine sites; toponym no. 11 or 12 may be Gezer, though there are alternate readings (Makkedah and Gaza respectively) [19, 21–27]. While the textual and archaeological evidence does not support viewing the Karnak relief simply as a list of sites attacked or destroyed by Sheshonq I [25], most scholars consider that a campaign into the southern Levant did occur, perhaps partly (or primarily?) aimed at disrupting or controlling the copper trade [28–30]. Sheshonq I is commonly equated with biblical “Shishak king of Egypt”, who is described as attacking Jerusalem in the 5^th^ regnal year of Solomon’s son Rehoboam (1 Kings 14:25–26; 2 Chron. 12:2–9) [19–32]. *If* the rulers are indeed equivalent, and an attack on Jerusalem historical, then Gezer likely also came under pressure since it guards the western end of the main route leading up to Jerusalem [32, 33].

The last major reference to Gezer during the Iron Age occurs in contemporary Assyrian sources: a siege of Gezer (Ga-az-ru) by Tiglath-pileser III, dated by textual evidence to 734 BC, is depicted in a palace relief at Nimrud [34–36].

### Archaeological excavation at Gezer

**Fig 2:**
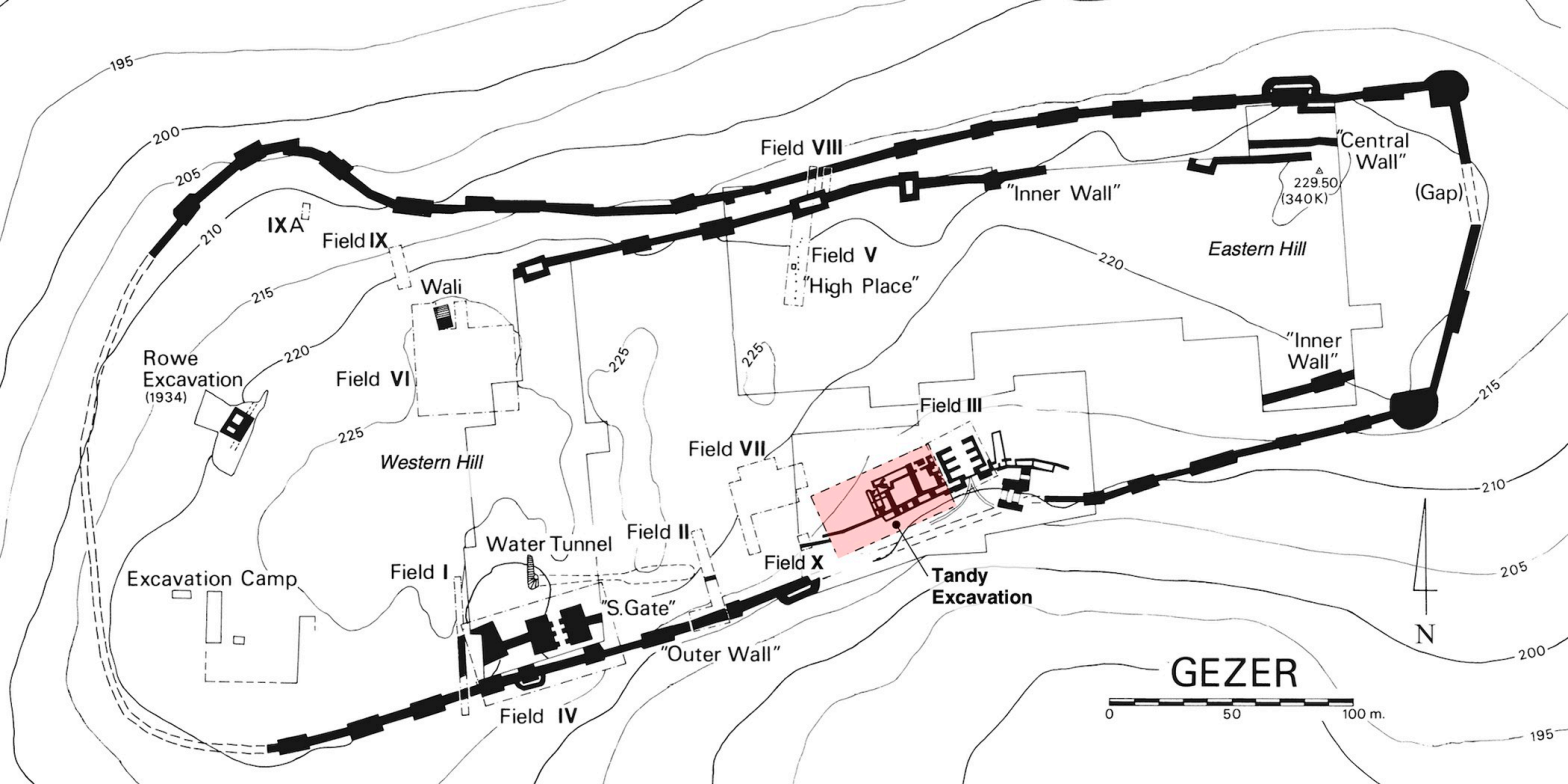
Location of the Tandy excavation relative to previous archaeological fieldwork at Gezer. Image adapted from [41] (front plan) under a CC BY license, with permission from J. Seger, original copyright 2013.

Gezer has been the subject of archaeological fieldwork for over a century, with many parts of the site investigated (Fig 2). Macalister was the first to excavate (1902–1909) [37], but his rudimentary excavation methods seriously limit our ability to integrate the findings into a reconstruction of the site’s history [38]. This is unfortunate, since he excavated nearly 60% of the tell–a fact that leaves few locations available to modern excavators. Nonetheless, Macalister exposed a number of key structures that should be associated with the Late Bronze and Iron Age cities. These include portions of city gates and fortification walls along the southern edge of the site, in the saddle area between Gezer’s western and eastern mounds [37, 39, 40].

Following projects of limited scope by Weill in 1912–1913 and 1923–1924 [42], and Rowe in 1934 [43–45], the next expedition to undertake extensive excavation, this time using careful stratigraphic methods, was by Hebrew Union College (HUC) under the direction of Wright, Dever, Lance and Seger between 1964 and 1974. Remains of the late LBA and Iron Age were explored particularly in Fields II [46, 47], Field III [48, 49], Field VI [50] and Field VII [51, 52]. In the saddle area, Field VII presented the most detailed Iron Age sequence, while Iron II fortification systems and part of an administrative building were explored in Field III. Exploration of features initially exposed but misdated by Macalister revealed six- and four-chambered city gates and a casemate wall.

Dever returned to Gezer for two additional seasons in 1984 and 1990 in an effort to clarify the date of the ‘Outer Wall’ and lower gateway, and to explore the Iron II administrative building west of the six-chambered gate in Field III [53–57].

**Fig 3:**
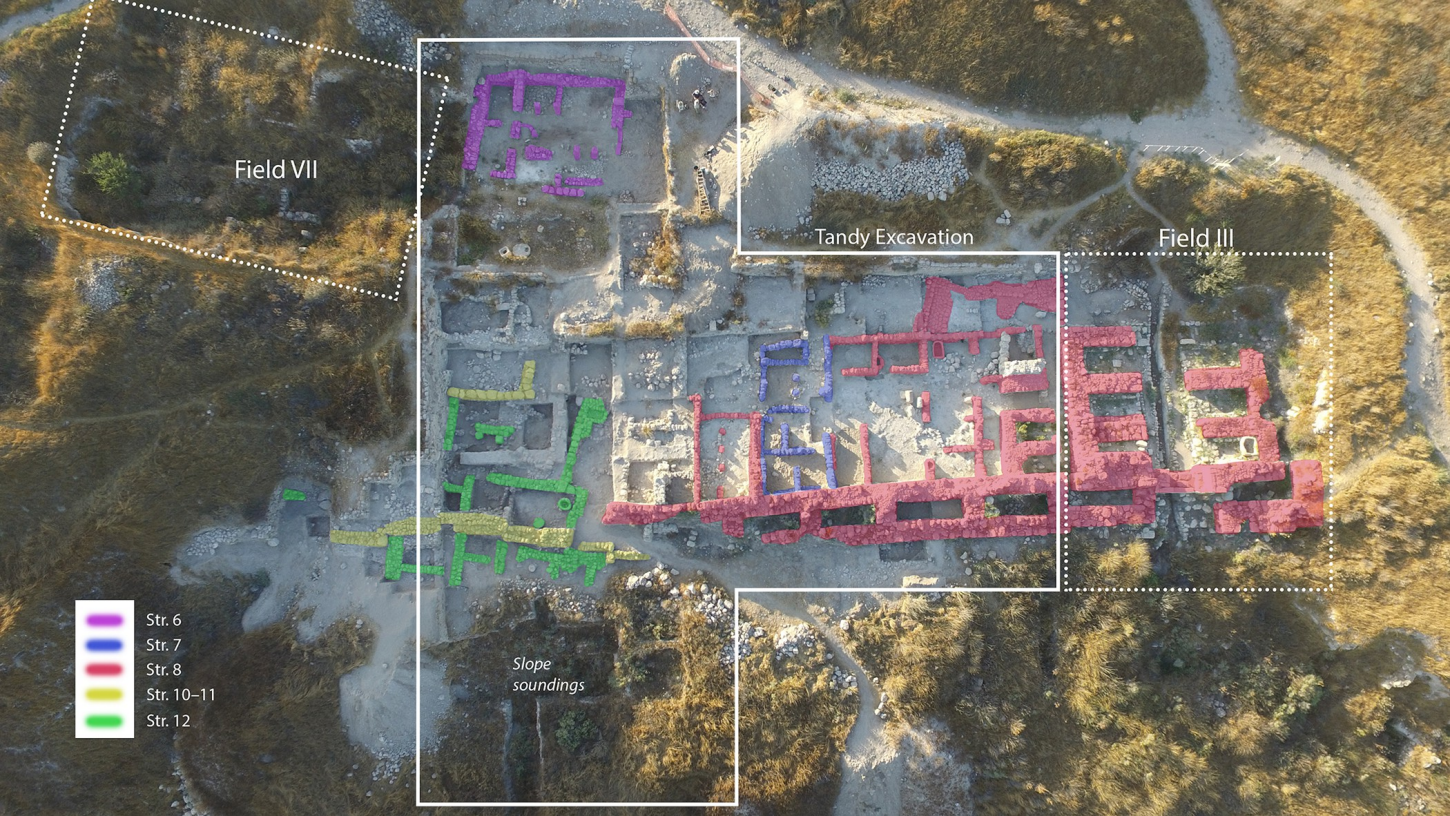
Aerial view of the Tandy excavations, with a wide exposure of iron age strata on the central-southern edge of the Gezer mound between fields VII and III of Hebrew Union College.

Fieldwork at Gezer was renewed between 2006 and 2017 by Ortiz and Wolff, focused on creating a wide exposure of the Iron Age city between HUC Fields VII and III (Fig 3). Ten seasons of excavation under the auspices of the Tandy Institute of Archaeology (Southwestern Baptist Theological Seminary) revealed continuous occupation through three strata of the late LBA to Iron I and four of Iron II (cf. [58–61], final publication in preparation) (Table 1). The plans attest to the changing nature of activity near the city gate–sometimes domestic and at other times administrative. Earlier periods (LBA–Iron I) were represented mainly in the western portion of the excavated area, and Iron II in the east. For convenience, the excavation project is referred to throughout this article as the Tandy expedition, but note that during the publication phase the project was moved to the Lanier Center for Archaeology at Lipscomb University.

**Table 1:** Stratigraphy of the Gezer Tandy excavation, with reference to HUC stratigraphy. Destruction horizons are marked red.

Tandy stratum	Cultural period	Description	HUC site-wide stratum
Overlying: Persian & Hellenistic remains (Strata 4 & 3) and later activity (Strata 2 & 1).			
6A	Iron IIB	Minor rebuilding and modifications.	VI
6B		Administrative buildings A-B, Industrial Building C, rebuilt fortification walls, Four Room House, courtyard and street. **HUC:** domestic buildings (Field VII) and re-used four-chambered gate.	
7	Iron IIA	Domestic units A–E, dog burials and re-used city wall. **HUC:** four-chambered gate.	VII
8	Iron IIA	Courtyard-type Administrative Building, monumental flagstone pavement, two domestic buildings, casemate city wall and single-line continuation, rebuilt glacis. **HUC:** six-chambered gate.	VIII
9	Iron IC/IIA	Rectangular building and courtyard, with tabuns.	
10A	Iron IB	Complex of building units, isolated walls beneath casemate, single-line city wall and glacis.	X–IX
10B		Complex of building units, city wall.	XI
11	Iron IA/B	Complex of building units, city wall (‘Philistine 2’ pottery appears).	XIII–XII
12A	Iron IA = LB III	Repairs, rebuild of 12B buildings.	XIV
12B	LB IIB	Elite building, various wall stubs.	XV
13	LB IIA	Two walls of a monumental building, mostly unexcavated.	XVI
-	LB IB	Unexcavated	XVII
14	MB III–LB IA	Walls and glacis, settlement unexcavated.	XVIII

From 2010–2018, an excavation by Warner, Yannai and Tsuk under the auspices of New Orleans Baptist Theological Seminary (NOBTS) and the Israel National Parks Authority (INPA) revisited Field IV and the adjacent water system. The main goal was to re-expose the water system (previously known to Macalister), clarifying its date (now considered MBA) and how it functioned [62, 63].

Throughout this article, site-wide strata are denoted with Roman numerals and those of single excavation fields with Arabic numerals. The latter refer to the Tandy Expedition except where otherwise specified.

Gezer’s archaeology has played a significant role in many debates related to the chronology of the southern Levant during the late LBA through Iron Age (Table 2). Key issues at Gezer that have remained unclear until recently include:

- The extent and date of destruction at the end of the LBA, which the excavators suggest may be associated with Merneptah. HUC attributed limited burnt remains and smashed pottery in Field II and large-scale trenching in Field VI (the acropolis) with Stratum XV and the end of the LBA [47, 50]; much clearer evidence has now come from the excavations of the Tandy expedition. Still, our ability to securely set the absolute date of the destruction and test the viability of potential historical correlations using solely pottery and finds is severely limited.
- The chronology of so-called ‘Philistine’ material culture [2, 64–72]. Gezer is not a core Philistine-related site, but characteristic pottery appears quite suddenly in Stratum XIII, making up 5% of the relevant pottery assemblage in Fields VI [50] and the Tandy excavation. It first occurs as Philistine 2 (Bichrome) ware, and no indisputable examples of Philistine 1 (Monochrome) are known ([73, 74] contra [75]). A single sherd of Philistine 1 has been identified in the Tandy excavation (S. Gitin, personal communication). Determining the absolute chronology of when ‘Philistine’ influence first reached Gezer is of considerable interest, since it could enhance our understanding of social interactions during the LBA to Iron Age transition.
- The date of the ‘Outer Wall’ and lower gateway to either the LBA or Iron Age [55–57, 76–82]. The existence or lack of a fortification system at Gezer during the LBA has been vigorously debated, and fortification during the early Iron Age was also unclear until the Tandy expedition.
- The date and political association of monumental building activity in Stratum VIII, with its casemate wall, six-chambered gate and large administrative building. This marked change at Gezer was traditionally dated to the 10^th^ century BC [49, 53–55, 59–61], the gate initially featuring in chronological discussions due to Yadin’s association of six-chambered gates at Gezer, Hazor and Megiddo with 1 Kings 9:15 and Solomonic building activity [39]. The now well-recognised wide distribution of such gates shows that the style was not restricted to a particular kingdom nor were they necessarily built at the same time [83, 84]. Following a low chronology for the Iron I to IIA transition, Finkelstein and others dated Stratum VIII to the 9^th^ century BC and suggested associating it with the northern Israelite kingdom under the Omride dynasty [81, 85–88]. Recent intense archaeological research in the Shephelah shows Gezer Stratum VIII to be part of a pattern indicative of political expansion. Various models have been proposed, and the phenomena is usually seen as the result of westward expansion by Judah or polities based in Jerusalem or the Benjamin plateau [89–93].
- The date and possible historical association for the destruction of Stratum VIII. HUC and the Tandy expedition placed the destruction in the second part of the 10^th^ century BC, drawing an association with Shishak / Sheshonq I. A low chronology scenario, on the other hand, would put the event well inside the 9^th^ century BC, and Finkelstein has suggested associating it with the ca. 830 BC campaign of the Aramaean ruler Hazael [75].

**Table 2:** Iron Age chronology of the southern Levant.

IRON AGE CHRONOLOGY			
Period transition	Generally accepted date (BC)		
LB II to Iron IA = LB III	ca. 1180		
Iron IA to Iron IB-C	ca. 1130		
Iron I to Iron IIA	*Conventional [94]* ca. 1000	*‘Modified’ [95]* ca. 980	*Low [87, 96]* ca. 920/900
Iron IIA to Iron IIB	ca. 830		
Chronology of ‘Philistine’ material culture			
	*High [64]*	*Middle [65, 66]*	*Low [67, 68]*
Start ‘Philistine 1’ / Monochrome	before ca. 1180 BC (late 13^th^ / early 12^th^ c. BC)	ca. 1180–1130	late 12^th^ c. BC
Start ‘Philistine 2’ / Bichrome	ca. 1180–1130	late 12^th^ c. BC	early 11^th^ c. BC

Note: Some scholars argue there was little or no Philistine 1-only phase prior to Philistine 2 [72, 97].

### Radiocarbon and Gezer

Over the past two decades, the radiocarbon method has contributed greatly to setting absolute dates for key late LBA and Iron Age stratigraphic sequences in the southern Levant and played an increasingly central role in attempts to resolve prominent chronological issues. Radiocarbon data has been published from key northern sites such as Dor [98], Megiddo [99–101], Beth Shean [102] and Rehov [103], in south-central Israel at Lachish [2, 91], Tel es-Safi [104], Tel Miqne [71, 98], Jaffa [105], Beth Shemesh [106, 107], Azekah [2, 108], Khirbet Qeiyafa [109, 110], Ashkelon [111] and Qubur el-Walaydah [112], and further south in the Negev and copper mining districts [30, 113–115]. Large datasets covering long, continuous stratigraphic sequences are best able to contribute to chronological debates, since prior knowledge of relative order can be used to constrain the radiocarbon probabilities using Bayesian statistics [116–118]. However, in southern Cisjordan many of the datasets are small and have major gaps; even at Lachish, which until now has offered the longest Iron Age sequence, there is a centuries-long gap during Iron I. Needless to say, the development of further robust datasets from continuous late LBA through Iron Age sequences–not least from large and historically significant sites such as Gezer–will prove crucial for solving chronological debates and accurately reconstructing the region’s history.

Until recently only a few ad-hoc ^14^C measurements were available at Gezer for any stratum or period [41, 47, 50, 51]. In cooperation with the Tandy and the NOBTS-INPA expeditions, we have now been able to develop detailed ^14^C datasets covering large parts of the MBA, LBA and Iron Age. Here we present a late LBA through Iron Age sequence from the largely continuous stratigraphic sequence revealed by the Tandy expedition.

There is understandably some reservation among scholars about making connections between archaeological remains and events, processes or individuals in textual sources, particularly given the tendency of past scholarship to accept these correlations rather hastily and uncritically. Nonetheless, we must cautiously compare the different lines of evidence available to us, bearing in mind their limitations–including the debated historicity of texts and open aspects of archaeological interpretation. Chronology is one key test for establishing the basic feasibility of potential archaeological-textual correlations; it can demonstrate possible contemporaneity of rulers/events and strata or it may show a correlation to be improbable or impossible. Indeed, one of the weaknesses of correlations proposed in past scholarship was the questionable precision or accuracy of traditional chronologies based on local and imported pottery/finds and textual evidence. The development of independent, robust chronologies through the application of radiocarbon dating and Bayesian modelling provides a major advance. Radiocarbon-based chronologies at key historical sites such as Gezer provide an opportunity to test the feasibility of new and previously proposed correlations. The aim is by no means to ‘prove’ that particular archaeological phenomena (including architecture and destruction layers) should be associated with a particular Egyptian ruler or biblical figure, as this would require more lines of evidence. Rather, we simply ask how well the correlations hold up from a purely chronological perspective.

### Stratigraphy of the tandy expedition

The earliest substantial remains exposed by the Tandy expedition are the MBA fortification and rampart (Str. 14; refer Table 1), exposed in soundings on the southern slope. However, the corresponding occupation for this period, and the early to mid-LBA, is unexcavated in this part of the site. The lowest occupation layer (Str. 13) has been uncovered in just one square and comprises two stone walls whose well-built masonry suggest a public building, tentatively ascribed to LB IIA.

**Fig 4:**
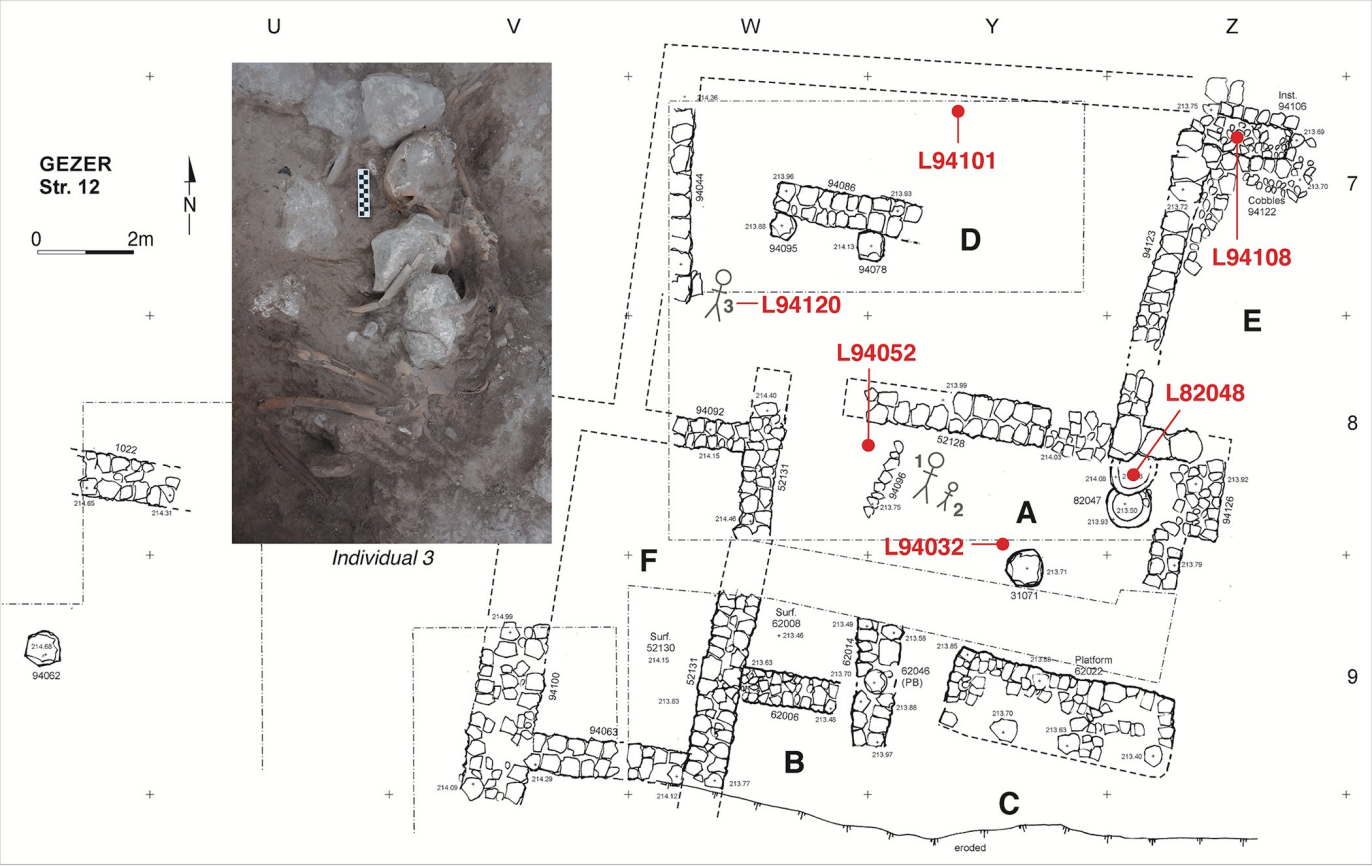
Plan of Tandy excavation stratum 12B elite residence with radiocarbon dated contexts marked. The insert shows Individual #3.

**Stratum 12B (XV; LB IIB)** is the earliest horizon for which a wide exposure has been achieved and features a large building (ca. 15 m x 20 m) (Fig 4). The building extends all the way to the slope edge and has partially eroded away, such that the southern closing wall can only tentatively be reconstructed. There are two major room units (A–C and D) and a courtyard (E); Room F forms an auxiliary western room or part of an adjacent building. Working Room A includes a stone vat and a well-worn disc-shaped working surface that was initially interpreted as a pillar base; small finds here included a scarab of Amenhotep III, a cylinder seal and several gold foil pieces. Several key finds were also retrieved from Room D, particularly a bifacial plaque with the cartouche of Thutmose III–a typical 19^th^ Dynasty product commemorating the 18^th^ Dynasty ruler. The overall nature and function of the Phase 12B building is uncertain, but the excavators suggest it served as an elite residency [58].

The fact that the Stratum 12B building extended to the slope edge, and that probes downslope failed to find a continuation of the Outer Wall (controversially dated by HUC to the LBA) may suggest that Gezer was unfortified during the LBA. Alternatively, a simple LBA city wall–perhaps doubling as the southern wall of the residence and adjacent buildings–may have eroded away from the slope.

The Stratum 12B building was destroyed in a fiery conflagration, whose calamitous nature is evidenced by the remains of three individuals. The badly burnt remains of an adult and child (Individuals #1, #2) were discovered on the floor of Room A, and an adult female evidently killed by the collapsing building was found in the southwest corner of Unit D (Individual #3; Fig 4 inset). Burnt destruction debris and restorable pottery were encountered in multiple rooms.

Stratum 12B was assigned to LB IIB based on pottery, the 19^th^ Dynasty bifacial plaque and the stratigraphic position below the distinctly Iron I Stratum 11. The destruction horizon has been associated with the campaign of Merneptah [58, 59].

In other excavation fields at Gezer only fragmentary in situ remains may be dated to LB IIB: Field I (local Str. 5) and Field II (local Str. 13) [46, 47]. Following new ^14^C dating in Field VI, we can no longer associate any in situ remains with the 13^th^ (nor 14^th^) centuries BC [2]. The destruction at the end of LB IIB may be represented in Field II (local Str. 13), where HUC exposed smashed storage jars and other vessels below a 25 cm layer of ash, charred beams and mudbrick debris. Widespread trenching in Field VI (local Str. 7) cannot be associated with this event.

#### Stratum 12A (XIV; Iron IA)

The inhabitants of Gezer evidently quickly re-established themselves following the destruction, as is indicated by a minor rebuild of the elite residency, and by similar re-use of architecture in Field II (local Str. 12) [47]. This phase is dated to Iron IA.

**Fig 5:**
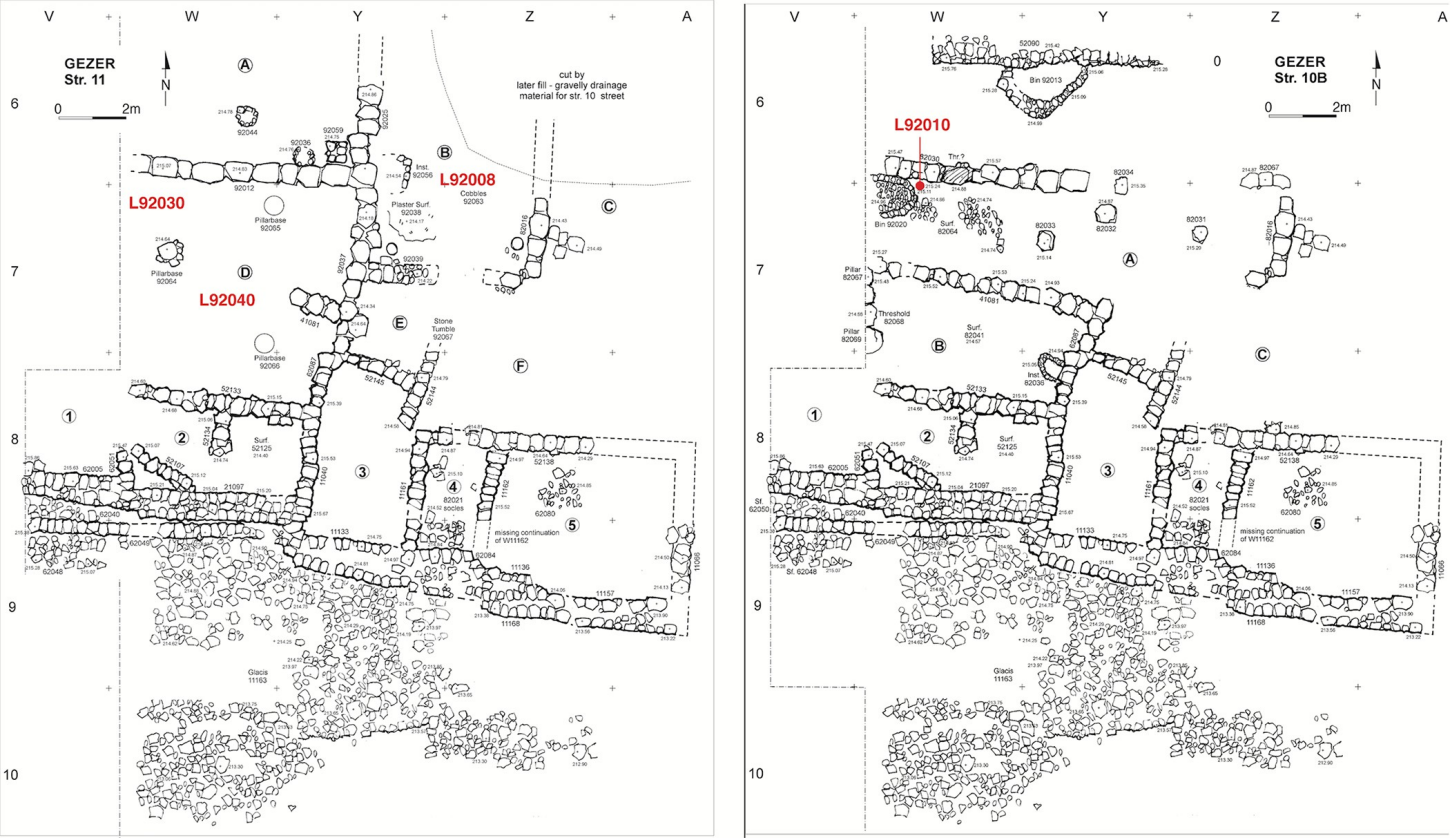
Plan of Tandy excavation strata 11 and 10B with radiocarbon dated contexts marked.

In **Stratum 11 (XIII–XII; Iron IA/B)** the layout changed completely (Fig 5 left). Gezer was apparently fortified during Iron I: a portion of the city wall was revealed along the edge of the slope, directly over the remains of Stratum 12. Little is known regarding the city gate or other elements of this fortification system. Against the northern face of the city wall, a row of irregularly sized units (1–5) was built, interpreted as storage rooms and perhaps forming a precursor to the Iron II casemate system. Further inside the city some 150 m^2^ of a building complex was exposed; this includes a large pillared room (D) and other partially-defined spaces to the north (A) and east (B, C, E, F). Stratum 11 has been dated to Iron IA/B. Notably, Philistine pottery (‘Philistine 2’ ware) appears for the first time in this horizon.

**Fig 6:**
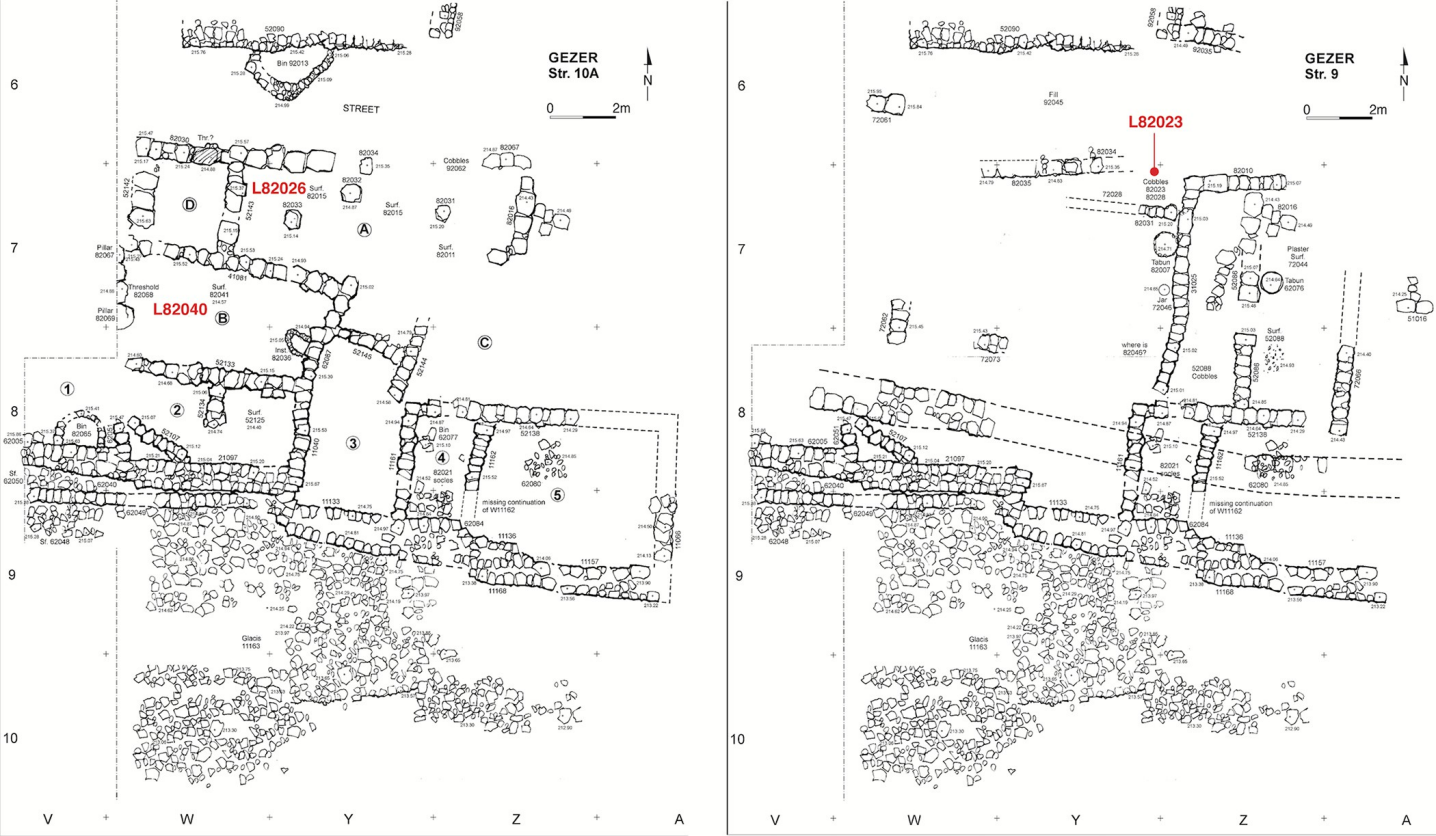
Plan of Tandy excavation strata 10A and 9 with radiocarbon dated contexts marked.

**Stratum 10 (XI–IX; Iron IB)** with its two sub-phases reflects modifications to the plan of the Stratum 11 complex (Fig 5 right). Surfaces were replaced and dividing walls added or removed until an arrangement of four spaces (A–D) was reached in Stratum 10A (Fig 6 left). In this last sub-phase an east-west street is evident, running along the northern wall of the complex. The fortification wall and row of units adjoining the complex to the south were used in Stratum 10 without modification.

Stratum 10A was violently destroyed, with evidence found in almost all rooms. The same event may be represented in Fields II (local Str. 7A), VI (local Str. 4) and VII (local Str. 8) [49, 50, 51]. There is no indication of intervening destruction events during Strata 11–10; HUC identified multiple Iron I destruction horizons only in Field VI, in Granary 24000 (local Str. 6) and the courtyard houses of local Stratum 5 [50].

Amongst the burnt debris of Stratum 10A, the Tandy expedition retrieved complete and restorable vessels characteristic of Iron IB. Room 3 of Stratum 10A yielded several mushroom-shaped clay stoppers, one of which bore a stamp seal impression that has been tentatively associated with the reigns of Siamun and Sheshonq I in the 10^th^ century BC [119, 120]. This seemed compatible with HUC’s proposed connection of the Stratum IX destruction with Siamun or another 21^st^ Dynasty ruler based on 1 Kings 9:16 [47].

**Stratum 9 (Iron I/IIA)** is an ephemeral phase that comprises the rebuild of a domestic structure (Fig 6 right). The builders were well aware of the destroyed Stratum 10 horizon and built directly on its architecture, integrating or reusing some architectural elements (e.g. Room 4 walls of Stratum 10). Stratum 9 seems to be associated with a new city wall that was subsequently further transformed in Stratum 8. The stratum may belong to late Iron I or early Iron IIA.

**Fig 7:**
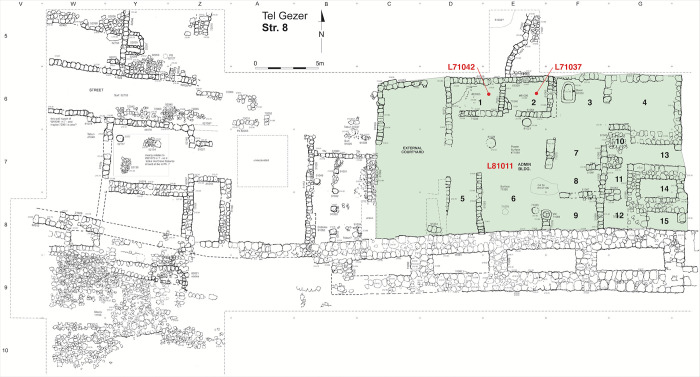
Plan of Tandy stratum 8 with radiocarbon dated contexts in the courtyard-type administrative building marked.

**Stratum 8 (VIII; Iron IIA)** signals a major transformation at Gezer, with the appearance of monumental architecture (Fig 7). A new fortification system featuring a massive six-chambered gate, casemate wall and new stone-covered glacis was built in this part of the site, and a large administrative building laid out close by. Macalister encountered the gate and casemate but misdated them to the Hellenistic period [37]. Both were investigated stratigraphically by HUC after Yadin identified the gate’s partial plan in Macalister’s drawings–similar to gates at Hazor (X) and Megiddo (Str. VA–IVB) [39, 48, 49]. The Tandy expedition re-exposed ca. 27 m of the casemate wall west of the gate (after which it gives way to a single wall line) and identified an accompanying stone-covered glacis. To the fortification system of Stratum 8, HUC would add the Outer Wall and lower gateway [55], but the stratigraphic associations of these elements is debated. Finkelstein [75] has associated the construction of both with Stratum VII (= Tandy Stratum 7).

Abutting the interior of the casemate wall and separated from the gate structure only by a narrow alley is the large Courtyard-type Administrative Building, of which a limited portion was excavated by HUC (as ‘Palace 10000’) [54]. Full exposure by the Tandy expedition revealed a ca. 19 x 12 m building with at least 15 distinct rooms/areas (Fig 7). The rectangular plan with small rooms arranged around a large central courtyard fits the Iron Age tradition of large administrative buildings (e.g. Megiddo Palace 6000) and echoes the so-called *bit hilani*-type palaces of the northern Levant. Buildings of this style in the southern Levant have been called Lateral-Access Podium (LAP) structures [121] or Central Hall Tetra-Partite Residencies [122]. The eastern part of the building (adjacent to the city gate) includes several thick-walled chambers that may have formed part of a defensive tower. Several rooms in the northwest feature neatly plastered floors, and ashlar masonry blocks formed a divider in the central courtyard. The administrative building is bordered to the north by a street and monumental stairway. Immediately to the west, the Tandy team exposed a large open courtyard, a small pillared building and several domestic building units built on either side of an east-west street.

Stratum 8 seems to have suffered a major destructive event. Most walls of the administrative building had fallen in the same direction (westward) and the structure was buried in up to 1.5 m of mudbrick debris; concentrations of boulders filled some rooms. The inhabitants seem to have been forewarned against an impending disaster, as the building was found largely empty. HUC found evidence of destruction in the adjacent six-chambered gate, as well as in Field VII [48, 49, 51]. Pottery from the Tandy and HUC excavations put Stratum 8 (VIII) firmly in Iron IIA.

**Fig 8:**
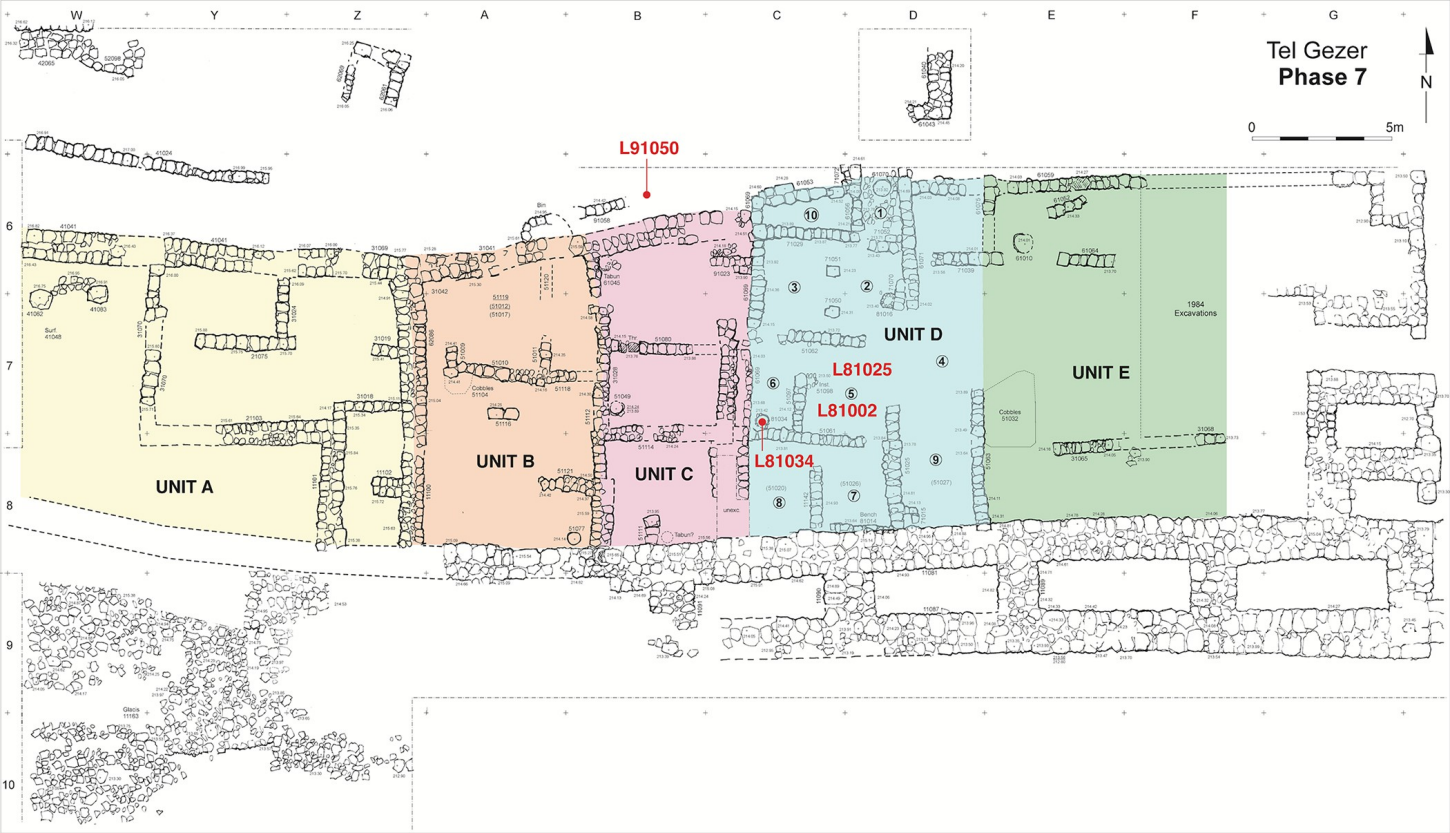
Plan of Tandy stratum 7 domestic units with radiocarbon dated contexts marked.

In **Stratum 7 (VII; Iron IIA)** the character of the gate area changed from administrative to domestic, and adjoining units were built along the north face of the city wall (Fig 8). One fully excavated building unit (D) includes a main pillared room and an interconnected group of storage and work rooms. The casemate wall was re-used but the gate was rebuilt with a four-chambered plan [48, 49].

Stratum 7 came to a sudden end, as evidenced by a destruction layer in the pillared unit that included a large assemblage of Iron IIA restorable vessels. The destruction was initially thought to date to the second part of the 9^th^ century BC and associated with the campaign of the Aramaean ruler Hazael ca. 830 BC, which destroyed the nearby city of Gath (2 Kings 12:17) and possibly other sites [60]; ceramic parallels were initially drawn with Tell es-Safi (Gath) and Tel Zayit.

In **Stratum 6 (VI; Iron IIB)** the character of the architecture changed again, and three major public buildings were erected west of the city gate. In the northwestern part of its excavation, the Tandy expedition exposed a Four Room House [123], similar to but significantly larger than others found in adjacent Field VII [51]. The domestic buildings show clear evidence of destruction by fire, and the end of the stratum has been associated with the historically well-known campaign of the Assyrian monarch Tiglath Pileser III [35, 60].

## Materials and methods

### Sampling

Short-lived organic materials (primarily charred seeds) suitable for radiocarbon dating were collected throughout the ten Tandy excavation seasons, found in association with floors, installations, phytolith layers, burials and destruction layers. During the 2017 field season, the lead author worked with the team to improve the retrieval rate of smaller seeds/fragments from the most secure contexts (i.e. low likelihood of residual or intrusive material) by using targeted fine dry sieving. Samples for dating were selected to represent the series of overlying occupation horizons, where possible using multiple contexts and at least three measurements per stratum. Priority was given to contexts with evidence of in situ burning or larger concentrations of organic material (seed clusters, phytolith layers). Almost the whole late LBA through Iron IIA stratigraphy, from Stratum 12B through 7 was addressed (Table 3), with data lacking only for Stratum 12A. We initially chose not to radiocarbon date Stratum 6 because its expected chronological position (destroyed in 734 BC) would place it on the Hallstatt Plateau [124].

**Table 3:** Radiocarbon dates from the Tandy excavation at Tel Gezer. All measurements were made on charred seeds, with the exception of SANU-60015 (bone collagen). Adjacent pairs of results marked with an asterisk (*) were measured on the same seed. hpd = highest probability density.

Yr/Locus	Basket	Botanical ID	Lab #	δ^13^C ± 1σ (‰)	^14^C Age ± 1σ (yrs BP)	Unmodelled Calibrated Age Range (BC) † 68.3% hpd	Unmodelled Calibrated Age Range (BC) † 95.4% hpd	Context Description
**Stratum 7 (Iron IIA)**								
15/81025	81222	Olea europaea	Beta-436536	-20.7 ± 0.3	2730 ± 30	900–888 (12.2%) 885–831 (56%)	929–809 (95.4%)	On surface / in floor matrix, Unit D Room 5.
15/81002	81044	Olea europaea	GrM-13324	-21.4 ± 0.12	2761 ± 15	924–897 (39.7%) 870–843 (28.6%)	972–955 (4.7%) 932–834 (90.8%)	In destruction debris on floor of Unit D Room 5.
	81077	Olea europaea	GrM-13325	-21.67 ± 0.12	2762 ± 15	925–897 (41.5%) 870–844 (26.8%)	973–955 (5.5%) 932–834 (89.9%)	
	81043	Olea europaea	OZX993*	-22.9 ± 0.2	2815 ± 20	999–993 (6.8%) 989–962 (29.5%) 959–930 (32%)	1041–1037 (0.8%) 1015–907 (94.7%)	
	81043	Olea europaea	OZZ478*	-23.0 ± 0.1	2850 ± 25	1050–977 (58%) 952–935 (10.2%)	1109–1064 (12%) 1059–927 (83.5%)	
15/81034	81237	Olea europaea	Beta-436537	-20.5 ± 0.3	2830 ± 30	1014–929 (68.3%)	1108–1095 (1.6%) 1082–1067 (1.9%) 1057–904 (92%)	Inside tabun (cluster of 20–30 olive pits), Unit D Room 6.
		Olea europaea	OZZ479*	-21.9 ± 0.4	2840 ± 25	1045–1031 (9.5%) 1018–971 (40.6%) 956–932 (18.1%)	1108–1095 (2.1%) 1082–1067 (2.5%) 1057–917 (90.9%)	
		Olea europaea	OZX994*	-22.7 ± 0.1	2845 ± 20	1047–1029 (14.3%) 1020–977 (41.5%) 951–935 (12.6%)	1107–1096 (1.8%) 1081–1068 (2.2%) 1056–923 (91.5%)	
16/91050	91237	Olea europaea	OZV267	-19.3 ± 0.1	2860 ± 20	1104–1100 (1.8%) 1077–1071 (2.8%) 1054–984 (63.7%)	1114–972 (88%) 956–933 (7.4%)	With dog burial, north of Unit C.
**Stratum 8 (Iron IIA)**								
15/71037	81030	Olea europaea	OZZ477*	-23.2 ± 0.1	2785 ± 25	982–946 (30.4%) 941–901 (37.9%)	1008–894 (83.9%) 877–839 (11.5%)	On floor of Room 2, northwest part of the administrative building.
		Olea europaea	OZX995*	-23.2 ± 0.1	2815 ± 25	1000–930 (68.3%)	1046–1030 (3.5%) 1019–903 (91.9%)	
	81028	Olea europaea	Beta-436539	-20 ± 0.3	2800 ± 30	998–993 (3.6%) 990–912 (64.7%)	1045–1033 (1.8%) 1017–895 (87.7%) 876–840 (6%)	
15/71042	81006	Olea europaea	Beta-436538	-20.5	2940 ± 30	1215–1111 (65.3%) 1090–1087 (1.3%) 1063–1059 (1.7%)	1257–1245 (1.9%) 1229–1046 (92%) 1029–1019 (1.5%)	On floor of Room 1, northwest part of the administrative building.
15/81011	81143	Olea europaea	Beta-436540	-21.4 ± 0.3	2980 ± 30	1261–1192 (46.2%) 1177–1159 (10.8%) 1145–1128 (11.2%)	1375–1353 (2.7%) 1300–1111 (91.8%) 1091–1085 (0.5%) 1064–1058 (0.5%)	Above courtyard surface in Room 6 of the administrative building.
**Stratum 9 (Iron IC/IIA)**								
15/82023	82105	Olea europaea	Beta-436533	-21.9 ± 0.3	2830 ± 30	1014–929 (68.3%)	1108–1095 (1.6%) 1082–1067 (1.9%) 1057–904 (92%)	On cobbled surface.
**Stratum 10A (Iron IB)**								
15/82026	82190	Olea europaea	Beta-436535	-22.1 ± 0.3	2900 ± 30	1125–1042 (56.9%) 1036–1016 (11.4%)	1210–1138 (19.4%) 1136–1005 (76%)	Cluster in destruction debris, together with smashed storage jars in Room A.
15/82040	82176	Olea europaea	Beta-436534	-22.7	2910 ± 30	1190–1179 (5.5%) 1157–1146 (5.9%) 1127–1047 (53.2%) 1028–1020 (3.7%)	1210–1012 (95.4%)	Concentration of olive pits on plaster surface of Room B.
		Olea europaea	OZZ480*	-20.3 ± 0.1	2895 ± 25	1119–1045 (58.2%) 1032–1017 (10.1%)	1201–1167 (6.8%) 1165–1142 (5.1%) 1131–1004 (83.5%)	
		Olea europaea	OZX996*	-22.1 ± 0.2	2945 ± 25	1212–1120 (68.3%)	1256–1247 (1.5%) 1227–1051 (93.9%)	
**Stratum 10B (Iron IB)**								
16/92010	92089	Olea europaea	GrM-10988*	-24.13 ± 0.05	2875 ± 18	1108–1095 (9.6%) 1081–1068 (9.8%) 1056–1010 (48.9%)	1122–984 (94.9%) 945–940 (0.5%)	Concentration of olive pits on cobbled surface L82064, next to bin L92020, Room A.
		Olea europaea	OZZ481*	-24.1 ± 0.3	2865 ± 25	1109–1092 (8.7%) 1084–1066 (9.5%) 1057–998 (49%) 993–990 (1.2%)	1121–970 (88%) 960–931 (7.4%)	
		Olea europaea	OZX997	-21.6 ± 0.2	2940 ± 25	1212–1115 (68.3%)	1255–1249 (0.8%) 1224–1049 (94.4%) 1025–1023 (0.3%)	
**Stratum 11 (Iron IA/B)**								
16/92040	92251	Olea europaea	GrM-10990*	-21.99 ± 0.05	2873 ± 18	1108–1095 (8.7%) 1081–1068 (9.1%) 1056–1009 (50.5%)	1121–983 (94.6%) 945–940 (0.8%)	Immediately above plaster surface in Room D, beside smashed vessel.
16/92040	92251	Olea europaea	OZV272*	-22 ± 0.1	2930 ± 25	1200–1168 (20.2%) 1167–1141 (17.5%) 1133–1108 (16.5%) 1095–1081 (7.4%) 1068–1056 (6.7%)	1218–1046 (93.6%) 1029–1019 (1.9%)	
		Olea europaea	OZV273	-22.1 ± 0.1	2955 ± 20	1214–1186 (23.5%) 1180–1154 (23.5%) 1148–1126 (21.2%)	1259–1243 (3.1%) 1232–1109 (88.5%) 1094–1081 (1.9%) 1068–1055 (1.9%)	
16/92030	92201	Olea europaea	OZV270	-22.7 ± 0.1	2990 ± 20	1263–1200 (62%) 1168–1166 (1%) 1141–1133 (5.2%)	1367–1359 (1.3%) 1284–1154 (84%) 1148–1126 (10.1%)	On plaster surface in Room D.
16/92008	92364	Olea europaea	OZV275	-21.2 ± 0.1	2990 ± 20	1263–1200 (62%) 1168–1166 (1%) 1141–1133 (5.2%)	1367–1359 (1.3%) 1284–1154 (84%) 1148–1126 (10.1%)	Lamp-and-bowl deposit in Room B, with several olive pits inside.
**Stratum 12B (LB IIB)**								
17/94108	94567	Olea europaea	GrM-13322	-22.62 ± 0.12	2961 ± 15	1216–1190 (25.9%) 1178–1157 (21.1%) 1146–1127 (21.3%)	1258–1244 (3.2%) 1231–1118 (92.2%)	Light grey ash contents of bin L94106 in Room E, rich in burnt bone and seeds
15/82048	82260	Olea europaea	Beta-436532	-22.3 ± 0.3	2970 ± 30	1257–1246 (6.1%) 1228–1155 (47%) 1148–1126 (15.1%)	1283–1105 (90.7%) 1101–1076 (2.7%) 1072–1054 (2.1%)	Debris inside Vat L82047, Room A.
17/94032	94556	Olea europaea	GrM-10986	-19.92 ± 0.05	2979 ± 17	1258–1244 (12.2%) 1231–1195 (34.5%) 1174–1162 (9.6%) 1143–1130 (12%)	1264–1153 (79.1%) 1149–1125 (16.4%)	Burnt destruction debris on floor in Room A, near working surface L31071.
17/94120	94656	Human bone, (collagen)††	SANU-60015	-18.55 ± 1	3005 ± 32	1370–1356 (5.9%) 1295–1202 (60%) 1140–1134 (2.4%)	1384–1340 (13%) 1313–1153 (75.9%) 1149–1125 (6.6%)	Unburnt fully articulated remains of Individual #3 on floor of Room D.
17/94052	94423	Triticum (free-threshing)	ETH-92839	-19.9 ± 1	2977 ± 22	1258–1244 (10.8%) 1231–1193 (32.3%) 1176–1160 (12%) 1145–1129 (13.3%)	1277–1120 (95.4%)	Dark seed-rich charred deposit found with restorable vessels on floor of Room A.
		Vitis vinifera	GrM-13319	-27.13 ± 0.12	3005 ± 15	1268–1216 (68.3%)	1374–1354 (5.3%) 1298–1197 (87%) 1172–1164 (1.5%) 1141–1132 (1.7%)	
		cf. Hordeum (humics)	GrM-13317	-23.11 ± 0.12	3092 ± 15	1408–1382 (29.4%) 1342–1309 (38.8%)	1419–1367 (41.9%) 1359–1295 (53.5%)	
17/94101	94607	Olea europaea	GrM-13321	-19.82 ± 0.12	3090 ± 15	1406–1382 (27.9%) 1342–1308 (40.4%)	1417–1367 (40.5%) 1360–1295 (54.9%)	Burnt contents of tabun in Room D.

† Results are rounded to single years. The significance of calibrated and/or modelled date ranges should, however, only be drawn in terms of decades, given the combined limitations of sampling, measurement, calibration and modelling.

†† Right humerus, compact bone from diaphysis. Adult individual. Acceptable preservation indicated by: collagen yield = 1.84%; %C = 42.87%; C:N = 3.19; δ^13^C = -18.55 ‰, δ^15^N = 9.62 ‰ [125].

Samples for Stratum 12B derive from a wide variety of contexts across the elite residency (Fig 4). Charred seeds were obtained from the burnt contents of a tabun (L94101; Room D), from ash-filled contents (L94108) of a bin (L94106; Room E) and burnt destruction debris on the floor of Room A (L94032, L94052). L94052 is a concentration of charred seeds found together with restorable pottery. Also associated with Stratum 12B are seeds obtained from Vat L82047 (contents L82048); destruction debris within the vat was generally unburnt, making the charred seeds from this context somewhat less secure. The unburnt fully articulated skeleton of Individual #3 (L94120) provided bone collagen for dating. Overall, the samples may be expected to represent the use of the building, predominantly its last years. No dateable material was obtained for the subsequent rebuild of the elite residency (Str. 12A).

Samples from Stratum 11 derive from organic-rich deposits spread across a plaster floor (L92030 and L92040; Room D), and from a foundation deposit (L92008; Room B) (Fig 5 left). Stratum 10B is represented by a concentration of charred olive pits (L92010) on cobbled surface L82064, immediately adjacent to bin L92020 in Room A; three measurements were made from L92010 (Fig 5 right). Stratum 10A samples come from seeds on the plaster floor (L82040) of Room B, and a seed concentration associated with storage jars in Room A (L82026; Fig 6 left). Just one sample was identified that can reliably date Stratum 9: seeds on cobbled surface L82023 (Fig 6 right).

From the Stratum 8 Courtyard-type Administrative Building, several charred seeds were found on the plastered floors of Rooms 1 and 2 (L71042 and L71037), and others above the courtyard surface of Room 6 (L81011) (Fig 7). Plentiful charred seeds for dating Stratum 7 were obtained from rooms in Unit D, most notably destruction debris (L81002) in Room 5 and the burnt contents of a tabun (L81034) in Room 6 (Fig 8). Additional seeds were found while dismantling the plaster surface of Room 5 (L81025) and within a dog burial north of Unit C (L91050).

### Radiocarbon dating

A total of 35 radiocarbon dates were run from seven strata / sub-strata, most represented by at least four measurements (Table 3). Multiple measurements were made for several contexts with large seed concentrations (e.g. Str. 12 17/94052, Str. 10B 16/92010, Str. 10A 15/82040 and Str. 7 15/81034). ^14^C measurement by Accelerator Mass Spectrometer (AMS) was carried out at five laboratories, primarily the Australian Nuclear Science and Technology Organisation (ANSTO) (15 dates), the University of Groningen (9 dates) and BETA Analytic (9 dates). All measurements were made on single entity charred seeds, except for one measurement on bone collagen (SANU-60015, Australian National University facility). Samples were pretreated using an Acid-Base-Acid (ABA) protocol to remove carbon-bearing contaminants [126, 127]; in one case the measurement was made on the humic acid component (GrM-13317). Following ABA, the bone collagen was extracted using a gelatinization and ultrafiltration protocol [128]. Pretreated samples were combusted and the resultant CO_2_ converted to graphite, a portion of which was used to determine δ^13^C by Isotope Ratio Mass Spectrometry (IRMS) for isotopic fractionation correction. AMS ^14^C measurement, along with standards and blanks, was made using a MICADAS (IonPlus®) accelerator at Groningen and ETH Zürich [129, 130], the ANTARES 10MV, STAR 2MV HVEE or VEGA 1MV NEC accelerators at ANSTO [131–133] and the Single Stage AMS at ANU [134].

Radiocarbon ages are reported in ^14^C years before present (BP) following international convention [135, 136]. Calibrated ages in calendar years were obtained using OxCal v 4.4 [118] and IntCal20 [137] interpolated to yearly intervals (Resolution = 1). Age ranges are given at 68.3% and 95.4% highest probability density (hpd; or ‘highest posterior density’ for modelled ranges).

Five ANSTO dates (prefix ‘OZX’) were revised by the laboratory after unstable current was noted in the AMS run, the correction giving younger ages by approximately 50 ^14^C years BP. This was validated by additional measurements (prefix ‘OZZ’) on the same seed or context.

### Bayesian modelling

To obtain more precise chronological information, radiocarbon data was combined with *a priori* knowledge of relative stratigraphic order using a Bayesian approach [116–118]. Using OxCal v 4.4, dates were arranged in a sequence of ‘phases’ according to the archaeological strata. No internal order was assumed between dates inside the same phase. Single boundaries indicate phases that are contiguous (i.e. strata thought to follow one another without a gap), while an extra boundary and empty phase were introduced where radiocarbon data is lacking (e.g. Stratum 12A). Between Phases 9 and 8 an extra boundary was used due to the especially marked change in architecture and possible gap, and the low data quantity (single date) representing Phase 9. Weighted averages were applied only for measurements from the same seed and only when these pass the χ^2^ test. Note that the Bayesian models use only radiocarbon data and stratigraphic order within the Tandy excavation field; no constraints from historical information were applied.

There are two approaches to addressing outliers in OxCal [138]. A common strategy has been to iteratively remove dates with the lowest agreement index from the model until the overall model agreement index exceeds 60%. This can sometimes result in the complete exclusion of a substantial portion of data. A second approach utilises OxCal’s outlier functionality to automatically identify and downweigh poorly fitting data. The probability of a date being an outlier is assumed to follow a Student’s *t* distribution (the so-called ‘General’ model) and an initial 5% *prior* probability is assumed. The model subsequently calculates *posterior* outlier probabilities for all dates based on the model fit. These assumptions are appropriate for short-lived materials, which comprise all the Tandy excavation radiocarbon samples. The second approach to outliers–which is the one primarily employed here–is preferable because it generally reduces the need to manually eliminate dates from models. It is, however, sometimes still prudent to test the effect of fully excluding those dates identified as probable outliers, to ensure they are not unduly influencing the model. For robust modelling, both approaches to outliers should yield very similar results. For the purpose of comparison we provide a model using agreement indices in the supplementary data.

## Results

### ^14^C data before modelling

**Fig 9:**
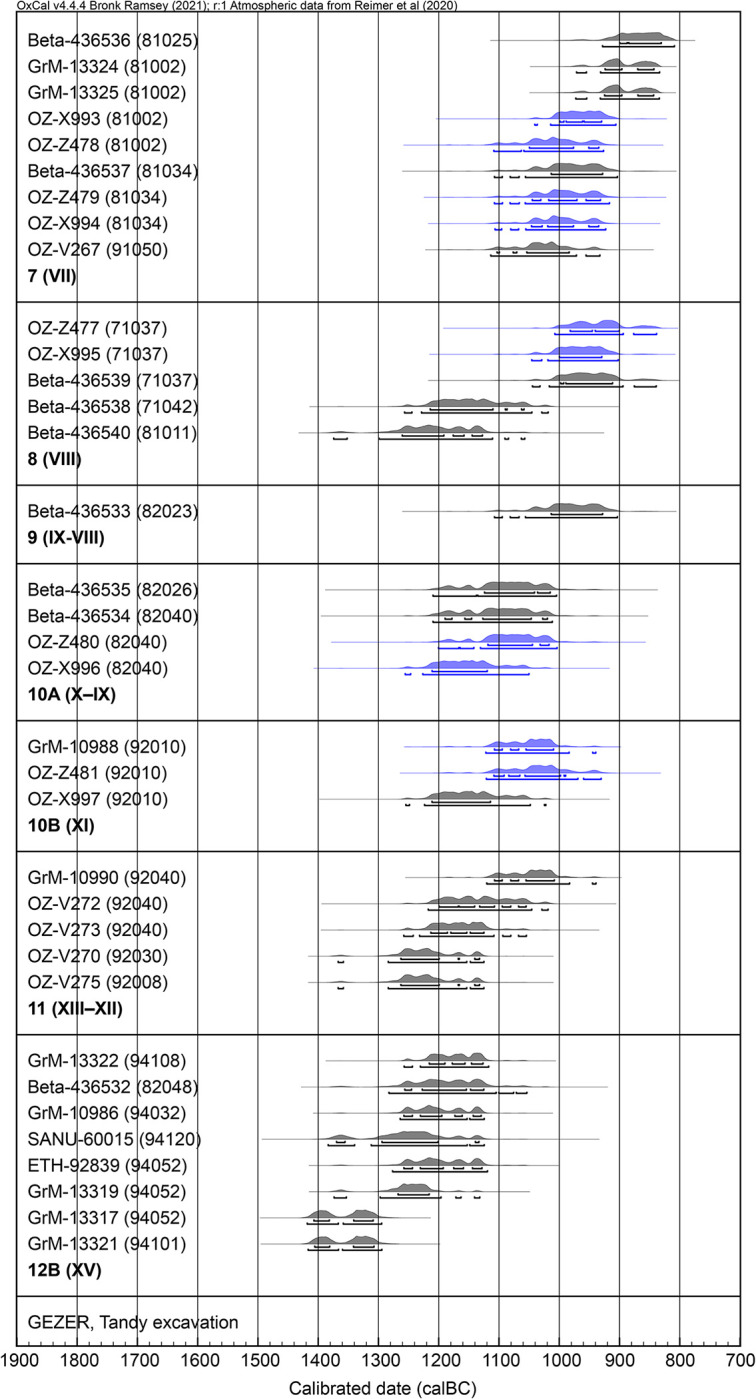
Independently calibrated ^14^C data from the Tandy excavation. Pairs of measurements from the same seed are marked blue. Highest probability density (hpd) ranges at 68.3% and 95.4% are marked with bars below each result.

The set of independently calibrated radiocarbon dates from Tandy Strata 12B–7 generally reflect the stratigraphic order well (Table 3, Fig 9). The great majority of results are consistent within each stratum/sub-stratum, bearing in mind effects due to the shape of the calibration curve. Dates from multiple laboratories and AMS runs show good agreement, including five pairs of measurements on fragments of the same olive pit (marked blue in Fig 9 and by an asterisk in Table 3).

Two dates in Stratum 8 (Beta-436538 and Beta-436540) seem to be outliers; these seeds were found close to surfaces but not in large clusters and hence the risk of residual material is higher. Two Stratum 12B dates (GrM-13317 and GrM-13321) also appear somewhat early (though with better overlap), but these samples are from large seed concentrations that were certainly burnt in situ and should be reliable; we suspect this may be simply a matter of measurement statistics, or the seeds include material from slightly earlier in the life of Stratum 12B.

### Bayesian modelling of ^14^C data with stratigraphy

**Fig 10:**
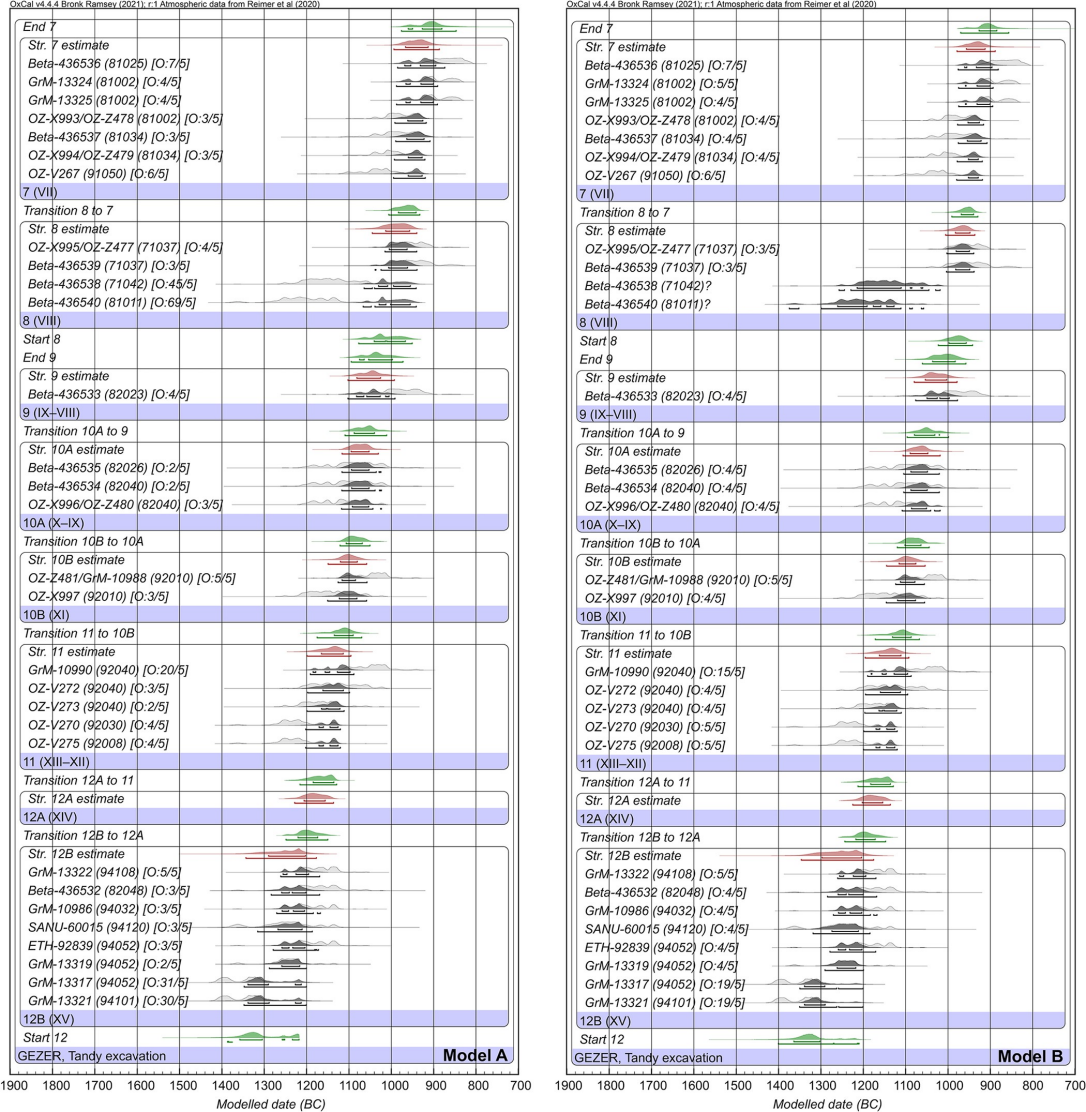
Bayesian ^14^C models (A and B) for the Tandy excavation. The models use OxCal’s outlier analysis. Model A includes all data, whereas Model B excludes two outliers in Tandy Stratum 8 (Beta-436538 and Beta-436540). Individual probability distributions before and after modelling are shown in light and dark grey respectively. Calculated transition boundaries are colored green, while date estimates for strata are red. Highest posterior density (hpd) ranges after modelling (68.3% and 95.4%) are marked with bars below each result. Prior and posterior outlier probabilities are indicated in square brackets after the laboratory number and locus. The OxCal code is provided in S1 Appendix.

Constraining the radiocarbon data with stratigraphic order using a Bayesian approach, Fig 10 Model A utilises all dates and applies OxCal’s ‘General’ outlier model with 5% prior outlier probabilities. The prior and posterior outliers are displayed for each date, after the laboratory code and locus. Only Beta-436538 and Beta-436540 of Stratum 8 show distinctly elevated posterior probabilities of being outliers (44% and 66% respectively). Since these two samples fit poorly with the surrounding data and their contexts are less secure (not seed clusters or burnt in situ, though found on floors), we opted to run a second version of the Bayesian model in which they are fully excluded (Fig 10 Model B). This does not have a major impact on model outcomes but does provide narrower and arguably more realistic estimates for Stratum 8 and its boundaries; hence we prefer this model.

For the purpose of comparison, a model that uses the alternate approach to outliers (i.e. agreement indices and manual, iterative removal of dates) is provided (Model C, S1 Fig). The results are very similar to Models A and B. To reach an overall model agreement index >60%, three dates were iteratively removed, including the same two Stratum 8 dates, and OZ-V267 of Stratum 7.

The output from all models is provided in S1 and S2 Tables, and the OxCal code in S1 Appendix. All elements of the models converged at ≥95%. In the following discussion, results are cited from our preferred model (B) unless specified otherwise. Table 4 summarises key results from Model B: phase transitions and use-length estimates, the latter obtained using OxCal’s ‘Date’ function.

**Table 4:** Estimated dates of strata and transitions. Results from preferred Model B are tabulated here. (For outputs from other Models refer to S1 and S2 Tables.) Shaded rows indicate where major cultural transitions occur. Calendar estimates for strata were obtained using the ‘Date’ function in OxCal, while transitions are represented by phase boundaries.

Cultural transitions	Stratum or transition	Modelled result, BC 68.3% hpd	Modelled result, BC 95.4% hpd
Iron IIA to IIB	End 7	927–885	970–857
	Stratum 7	957–913	979–889
	Transition 8 to 7	969–940	991–930
	Stratum 8	983–949	1006–937
	Start 8	998–957	1023–942
Iron I to II	End 9	1037–983	1061–958
	Stratum 9	1054–1003	1080–979
	Transition 10A to 9	1080–1021	1097–999
	Stratum 10A	1090–1048	1107–1019
	Transition 10B to 10A	1102–1064	1120–1045
	Stratum 10B	1116–1077	1145–1054
	Transition 11 to 10B	1131–1088	1172–1068
Iron IA to Iron IB	Stratum 11	1162–1112	1196–1093
	Transition 12A to 11	1183–1136	1213–1130
	Stratum 12A	1203–1155	1225–1137
LB IIB to Iron IA	Transition 12B to 12A	1218–1172	1244–1148
	Stratum 12B	1298–1204	1347–1176

The Bayesian models place Stratum 12 in the 13^th^ century BC, although we cannot yet reliably ascertain when this occupation horizon began. This must await further excavation, particularly of underlying Stratum 13. The end of Stratum 12B is more easily ascertained: constrained with the help of overlying strata, the destruction of the elite residence is placed 1218–1172 BC (68.3% highest posterior density, hpd). The subsequent rebuild of the residence (Str. 12A), though lacking direct data, evidently belongs to the first half of the 12^th^ century BC, and soon gave way to the completely new architecture of Stratum 11 (Start, 1183–1136 BC, 68.3% hpd). Stratum 11 characterised the second half of the 12^th^ century BC, with the modifications of Stratum 10 continuing into the first part of the 11^th^ century BC (10B: 1116–1077 and 10A: 1090–1048, 68.3% hpd). The destruction of Stratum 10A is estimated at 1080–1021 BC (68.3% hpd).

Intermediary Stratum 9, though represented by just one direct data point, is essentially constrained to the second part of the 11^th^ century BC. The transformation of Gezer in Stratum 8, with the erection of fortifications and the Courtyard-type Administrative Building, likely began in the early part of the 10^th^ century BC (998–957 BC, 68.3% hpd). If the two outliers Beta-436538 and Beta-436540 are included in the model (i.e. down-weighted rather than omitted), the start boundary of Stratum 8 includes the late 11^th^ century BC (Model A: 1041–967, 68.3% hpd). Stratum 8 was used during the first part of the 10^th^ century BC, until its destruction near the middle of the century (969–940 BC, 68.3% hpd). While we would ideally like to have additional radiocarbon dates for Stratum 8, the chronological position of this horizon is hard to dispute thanks to constraint provided by overlying Stratum 7.

Stratum 7, with its shift to domestic architecture in the gate area, was used primarily during the later part of the 10^th^ century BC. It was not particularly long-lived, as the site once again fell prey to a destructive event near the close of the 10^th^ century BC or early decades of the 9^th^ century BC (927–885, 68.3% hpd).

### Potential effect of radiocarbon offsets

The existence and effect of small radiocarbon offsets relative to the calibration curve have recently been a focus of investigation [139–143]. These can arise due to differences in region and growing season of the dated sample compared to the northern hemisphere tree data underlying IntCal; a further contribution can also come from measurement factors (e.g. AMS versus decay-counting). How the regional and growing offsets varied through time is not yet well understood, but all evidence indicates they are small in magnitude, around one to two decades at most. S2 Fig provides a test case whereby a hypothetical offset of 19±5 years is applied to Model B, by using the Delta_R function to shift dates before calibration. 19±5 years is likely an over-estimate, noting that most measurements were made on olive pits, which have a similar (summer) growing season to the northern hemisphere trees underlying IntCal20. The effect of such an offset on the Gezer results is modest, shifting results later by not more than a few decades.

## Discussion

The new radiocarbon series from the Tandy expedition allows us to better establish the absolute chronology of Gezer from the close of the LBA through Iron Age IIA. Since relatively few sites in this region were continuously occupied during the LBA to Iron Age transition (and even fewer of these are well-dated with radiocarbon), a key contribution is made to understanding the archaeology of the Shephelah and coastal plain. Gezer’s strategic position and frequent appearance in textual sources, and the availability of a radiocarbon-based chronology for Egypt, provides a rather unique opportunity to re-examine the impact on Gezer of the complex political changes that occurred in the region during the LBA to Iron Age transition. Bearing in mind the limitations of texts and questions of historicity, we may use the independent radiocarbon chronology of Gezer to test–from a strictly chronological point-of-view–the viability of proposed direct correlations between archaeological remains and recorded events or phenomena.

**Fig 11:**
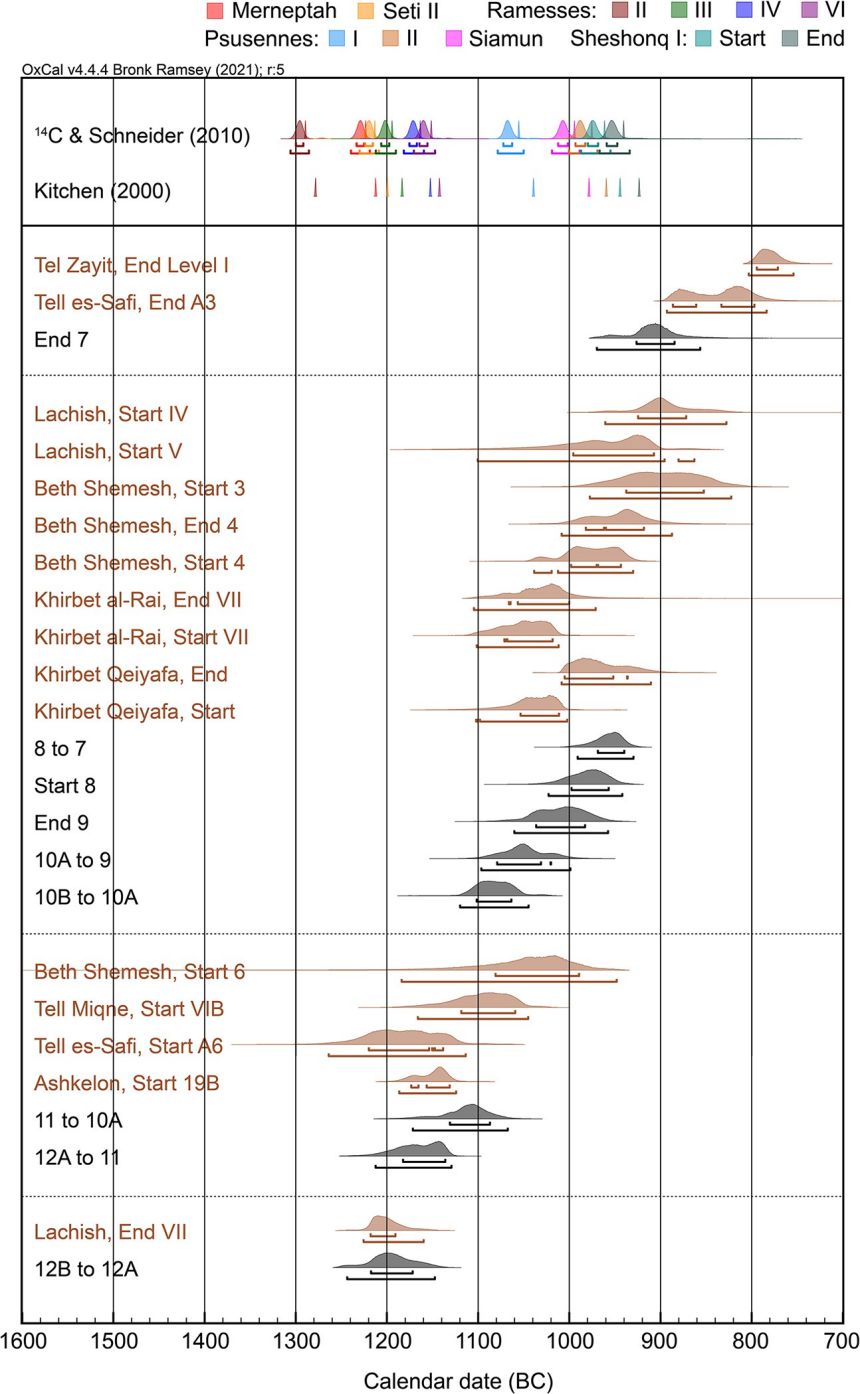
Comparison of ^14^C dated transitions at Gezer with key data from nearby sites and the Egyptian chronology. The ^14^C-based Egyptian chronology follows Dee [3] and Manning [4] and is updated with IntCal20 [137]. It assumes the ultra-high reign lengths of Aston [144] for Thutmoses III through Ramesses II and reign lengths from Schneider [145] for all others. Absolute date estimates based on traditional methods are shown for Schneider (same line as the ^14^C-based estimates) and Kitchen [146] (separate line below). Key results from sites neighboring Gezer are colored brown. Source models and data references for these sites are provided in S1 Appendix.

Fig 11 summarises the ^14^C-based dates of stratigraphic transitions at Gezer (using the preferred Model B), plotting them alongside radiocarbon results from other southern Levantine sites as well as the ^14^C-based and traditional accession dates for Egyptian rulers Ramesses II through Sheshonq I. Note that the New Kingdom model follows Dee [3] and Manning [4] and has been updated with IntCal20 [137]. Combining radiocarbon data with known regnal order and lengths (but no traditional absolute data), the model assumes Aston’s ultra-high reign lengths for Thutmoses III through Ramesses II [144], and reign lengths from Schneider [145] for all other rulers. ^14^C-based results from other southern Levantine sites were obtained using single-site models, for which OxCal code and data references are provided in S1 Appendix.

Radiocarbon data from the Tandy excavation confirms that Gezer was continuously occupied from the 13^th^ through 10^th^ centuries BC, despite multiple disruptive events and rebuilding episodes. The elite residency of Stratum 12B, with its signs of wealth and links to Egypt, provides a window on Gezer during the 13^th^ century BC that is currently only available in this part of the site. The sudden and fiery destruction of this building, in which multiple individuals were killed, occurred in the timeframe 1218–1172 BC (68.3% hpd; or 1244–1148 at 95.4% hpd). The impact of the event on the rest of the town is uncertain, though it may also have left traces in Field II. Various human or natural causes could be invoked to explain the destruction, but we note that the date is compatible with Merneptah’s campaign and his claim to have conquered Gezer (Fig 11). The ^14^C-based Egyptian model puts his accession at 1241–1219 BC (95.4% hpd) and that of Seti II at 1232–1209 (95.4% hpd). (Models using other reign length assumptions yield similar or slightly higher accession dates for these rulers.) Using the traditional approach to Egyptian chronology, Kitchen would date Merneptah’s reign at 1213–1203 BC [146], while Schneider would place it at 1224–1214 BC [145]. Applying a hypothetical offset of 19±5 year tends to weaken the fit between the Stratum 12B destruction (1198–1152 BC, 68.3% hpd; 1213–1131, 95.4% hpd) and Merneptah, whose reign in the Egyptian ^14^C-based model does not change by more than a few years.

The destruction of Stratum 12B fits well with radiocarbon data for destructive/disruptive events at other sites in the southern Levant [2]. It is notably similar to the destruction of Lachish–another major LBA city-state just 33 km to the south. Lachish Level VII shows evidence of widespread destruction and is well-dated by radiocarbon to 1218–1191 BC (68.3% hpd; Fig 11) [2]. While the direct causes of destruction or disruption at individual sites probably varied, and the events may have been separated by some years, the overall pattern is commonly viewed as part of a period of turmoil (the so-called ‘Crisis Years’) that affected the wider eastern Mediterranean region [147, 148]. Merneptah’s campaign and attempts to retain control of the southern Levant, appear to be a response to city-states and towns who were rebelling against Egyptian rule. Lachish is not mentioned by Merneptah but given its importance and proximity to Gezer we may speculate that it joined the rebellion or was targeted for its loyalty to Egypt; the latter is perhaps suggested by the strengthening Egyptian influence evident in the architecture and material culture of subsequent Level VI [149].

Gezer evidently recovered quickly with the rebuild of Stratum 12A in the first part of the 12^th^ century BC. The site’s status within the next (last) phase of Egyptian rule in the southern Levant is uncertain, but it does not appear to have had the elevated status of sites like Lachish (VI) and Azekah, which show accumulating wealth and strengthened ties with the Egyptian administration [150]. Perhaps for this reason, Gezer did not share the fate of Lachish and Azekah, which suffered impressive site-wide destructions in the second part of the 12^th^ century BC, after which they were abandoned for more than a century [2, 91, 108, 150, 151].

The Tandy excavation shows a substantial re-organization and planning of the city quarter and construction of a city wall in the timeframe 1183–1136 BC (68.3% hpd, Start Stratum 11). In this new setting, we see the arrival of so-called ‘Philistine’ pottery at Gezer, as type 2 / bichrome. Assuming the ware was indeed associated with the founding and main use of Stratum 11, the radiocarbon result suggests this pottery may have reached Gezer around the mid-12^th^ century BC. It was almost certainly present by the last decades of the century (Stratum 11 phase estimate: 1162–1112 BC, 68.3% hpd).

Gezer provides one of the most robust ^14^C-based estimates currently available for Philistine 2 pottery and its first occurrence at the borders of Philistia. The result closely matches recent ^14^C results from Ashkelon (Start 19B: 1173–1131 BC, 68.3% hpd) [111] and is compatible with Tell es-Safi (Gath) (Start A6: 1220–1138 BC, 68.3% hpd) (Fig 11) ([104], see analysis in [2]). Available data from Tel Miqne (Ekron) and Beth Shemesh give distinctly lower estimates for the strata in which Philistine 2 pottery first appear: Start Miqne VIB at 1118–1059BC and Start Beth Shemesh 6 at 1081–989 BC (both 68.3% hpd). However, a close review (for details, see [2]) suggests that the discrepancy may be due to data limitations: key strata at Tel Miqne are represented by single contexts [98], and measurements for Level 6 at Beth Shemesh come from a single olive pit [152].

Occupation at Gezer continued well into the 11^th^ century BC. There are indications of multiple disruption episodes in other parts of the site that are contemporary with Strata 11–10, notably Field VI (local Str. 6 ‘Granary’ and local Str. 5 Courtyard Houses) [50]. Unfortunately, these lack ^14^C data, but they may reflect Gezer’s position during Iron I, in a border/conflict region between emerging polities. In the southern part of the Gezer mound, destruction came in the timeframe 1080–1021 BC (68.3% hpd; or 1097–999 at 95.4% hpd), with the end of Tandy Stratum 10 (Stratum IX). This event seems to have been site-wide, reflected in nearby Field VII, local Str. 8 and perhaps also Field VI, local Str. 4.

HUC’s correlation of Stratum IX with Solomon’s era or Siamun, judged solely from the chronological point-of-view, seems improbable. The end of Tandy Stratum 10A is estimated by ^14^C within the 11^th^ century BC, contemporary with the 21^st^ Dynasty of Egypt but too early for Solomon by any estimate. There is limited overlap with the ^14^C-based estimate for the accession year of Siamun (1019–977 BC, 95.4% hpd) and none with traditional estimates for his reign (978–959 BC [146] or 995–976 BC [145]). Aside from any specific historical association at Gezer, the Tandy ^14^C results indicate that stamp seal impressions of the type found in the destruction can predate the reigns of Siamun and Sheshonq I. Indeed, the latest analysis of these seals associates them more broadly with the 21^st^ Dynasty (i.e. 1110–945 BC, 95.4% hpd by the ^14^C Egyptian chronology) [153].

The construction of Stratum VIII (Tandy Stratum 8) likely occurred in the first part of the 10^th^ century BC (Start 8: 998–957 BC, 68.3% hpd; 1023–942 BC, 95.4% hpd). The data and model–with constraints provided by overlying Stratum 7 –rule out a 9^th^ century BC date for Stratum VIII (contra [81, 85–88]). The start of Stratum 8 provides an estimate for the Iron I to IIA material culture transition in this geographic area. The transition cannot be later than Stratum 8, since this horizon is unambiguously Iron IIA, however it could (at least in theory) be slightly earlier since the attribution of intermediate Stratum 9 may be Iron I or Iron IIA. The results for Strata 9 and 8 fit acceptably with ^14^C results from transitional Iron I/IIA strata at other sites in the same region: Khirbet Qeiyafa [91, 109, 110], Khirbet al-Rai (Level VII) [91] and Beth Shemesh (Level 4) [106, 107]. Note that the boundaries shown in Fig 11 were calculated using independent single-site models that do not equate strata *a priori* based on pottery (cf. [154]). The earlier dates for the Iron I to IIA transition emerging from southern sites stand in contrast to later estimates from sites in the north such as Dor [98] and Rehov [103], suggesting the need for a more nuanced approach to the chronology of this period that explores potential delays in material culture change in different parts of the country.

The Iron I/IIA transition sees the onset of monumental buildings and fortifications indicative of central administration and the development or expansion of polities. Notably, radiocarbon shows that the phenomena appeared at Gezer and Khirbet Qeiyafa in a similarly early timeframe: late 11^th^ or early 10^th^ century BC. The start of Khirbet Qeiyafa should be treated somewhat cautiously since this is a single occupation horizon (thus with less constraint available for modelling) and most of the dates likely pertain to the later part of the stratum; nonetheless the founding of this well-fortified site, like Gezer, cannot date beyond the first part of the 10^th^ century BC. Other ^14^C-dated strata with indications of central administration may be somewhat later. The nature and fortification of Lachish V is disputed [91, 155–157] and its start date is not well-defined by ^14^C, but probably falls in the 10^th^ century BC [2, 91]. More definitive evidence of central administration at Lachish (Level IV) and Beth Shemesh (Level 3) is ^14^C-dated to the second part of the 10^th^ or first part of the 9^th^ century BC (Fig 11). The radiocarbon evidence thus suggests a prolonged process of expansion in the Shephelah.

The Shephelah region during the Iron I and Iron IIA is generally seen as a ‘middle ground’ between coastal (‘Philistine’) and highland polities [158]. Bunimovitz and Lederman define this region as a buffer zone that experienced “alternating prosperity and decline” [159]. Some scholars have discussed ‘Canaanite resistance’ [160] or a ‘Canaanite enclave’ [90] in the Shephelah, though identities are speculative and hard to access archaeologically; indeed, at a border site such as Gezer, identities or political alignments may have changed multiple times. To explain the growth in settlements and appearance of sites with monumental architecture and fortifications during Iron IIA, various models have been proposed: a westward expansion of a nascent Judah [89–91, 161] or another polity based in Jerusalem or the Benjamin Plateau [92, 93], formation of localised chiefdoms [162], the economic influence of the strong coastal (‘Philistine’) site of Gath [163], or a combination of these factors. The 10^th^ century BC ^14^C-based date for early expansion in the Shephelah notably rules out an association with the northern Israelite Omride dynasty (contra [88]), however it is chronologically compatible with Saul, David and/or Solomon, whose text-based dating (albeit approximate) falls in the 10^th^ century BC (perhaps also the late 11^th^ century BC). While scholars can debate the degree to which the accounts of these early highland rulers reflect historical memories, extra-biblical evidence indicates they were real historical figures [164–166], and most scholars see an early historical foundation to the later narrative development of the texts [167–172].

We propose that Gezer Stratum VIII represents a shift in political alignment of the city, corresponding to current models of state development in the region during the Iron Age IIA. (For a recent summary of the various theories of state development see [173]). The Tandy excavation directors consider that the most logical historical reconstruction based on the archaeological remains and ^14^C dates is the westward expansion of a nascent Judah already in the 10^th^ century BC (cf. [174] and [175] which confine Judah’s expansion to the 9^th^ century BC). The refined dating of Stratum 7 demonstrates that the Aijalon Valley was still a contested area at the end of the 10^th^ century BC and that the polity represented by the Stratum 8 monumental city was short-lived.

Stratum 8 came to an end already in the mid-10^th^ century BC (969–940 BC at 68.3% hpd; or 991–930 BC at 95.4% hpd). Radiocarbon suggests that Khirbet al-Rai and perhaps also Khirbet Qeiyafa were destroyed before Gezer Stratum 8 (Fig 11), consistent with the pottery evidence. The pottery assemblages at Khirbet Qeiyafa and Khirbet al-Rai are classified by the excavators as early Iron IIA [176, 177] but by other scholars as Iron I [178, 179] or transitional Iron I/IIA [154].

A comparison of the Stratum 8 destruction with the traditional and radiocarbon-based chronologies of Sheshonq I shows that this stratum may have come to an end during his reign (Fig 11). A ^14^C-based estimate for Sheshonq I puts his accession at 988–945 BC (95.4%) and the end of his reign at 967–934 BC (95.4%). (The traditional Egyptian chronology puts Sheshonq I’s reign at 945–924 BC [146] or 962–941 BC [145].) We do not, however, reach good agreement between ^14^C-based dates for Stratum 8 and Sheshonq I on the one hand, and the commonly cited historical-biblical date for Shishak’s campaign on the other: 925 BC based on Rehoboam’s 5^th^ year and synchronisms between later Israelite/Judahite reigns and Assyrian chronology. The discrepancy is modest, however: <10 years at 95.4% and <20 years at 68.3%. This is insufficient to rule out a convergence of the Egyptian sources, the Bible and radiocarbon, since we are conscious that:

1. There may well be leeway of 10–20 years for the Stratum 8 to 7 transition, given the limited number of Stratum 8 measurements.
2. There is room to debate the biblical date for Shishak’s campaign. Estimates for the 5^th^ year of Rehoboam are generally placed between ca. 930 and 915 BC [180–183] but one could potentially argue for dates as high as ca. 970 BC [184].
3. Sheshonq I is located at the tail end of the New Kingdom radiocarbon model. The quantity of ^14^C data for the 21^st^ Dynasty is limited (13 dates, of which 9 are from one reign: Amenemnisu) and small inaccuracies in New Kingdom reign lengths (often based on maximum attested regnal years) could cumulatively pull this reign too high.

The ^14^C-based results from Stratum 7 open another possibility for correlation with Shishak / Sheshonq I. The end boundary (927–885 BC, 68.3% hpd) includes the common biblical date for Shishak’s campaign, but does not fit well with current ^14^C-based estimates for Sheshonq I. In any case, the previously held historical association of Stratum 7 with the Aramean ruler Hazael in the second part of the 9^th^ century BC is firmly ruled out. The end boundary does not include the highest historical date for the campaign (ca. 830 BC) even at 95.4% hpd (970–857 BC). Comparison with ^14^C data at other sites shows that the event is unlikely to be contemporary with destructions at Tell es-Safi (Gath) and Tel Zayit Level I (Fig 11). There is minimal overlap at 68.3% between the end boundaries of Stratum 7 and Tell es-Safi Level A3, and none with Tel Zayit Level I even at 95.4% hpd. Unlike the situation at Gezer, the ^14^C evidence at Gath–the only city specifically mentioned in 2 Kings 12:17 as having been attacked by Hazael–converges well with Hazael’s campaign: a simple Bayesian model places the end of Tell es-Safi A3 at 887–798 BC (68.3% hpd). The Tel Zayit Level I destruction dates as much as a century after Tandy Stratum 7, perhaps even later than Hazael’s reign (end of Level I: 796–772 BC, 68.3% hpd). These outcomes raise caution concerning the tendency in scholarship to tightly group destruction layers based on pottery and historical sources; in reality these events may be associated with a wider variety of conflicts (recorded and unrecorded) and spread over a longer period of time. For Tandy Stratum 7, we ought to consider other skirmishes between Judah, Israel and their neighbors during the late 10^th^ and early 9^th^ centuries BC (e.g. 1 Kings 15:16–22), as well as non-military causes.

Gezer does not seem to have suffered any major destruction between ca. 900 BC and the second part of the 8^th^ century BC. The town may have been much reduced in size and importance during this time and, given the earlier-than-expected date of Stratum 7, we should consider whether there was an occupation gap (at least on the southern edge of the site) between Tandy Strata 7 and 6.

For the date of Stratum 6 we must rely for now on the evidence of the pottery and finds [35]. In view of the higher-than-expected date for Stratum 7, adding ^14^C data for Stratum 6 may in fact be worthwhile, helping to assess its founding date and confirming that the destruction did not occur substantially before 734 BC (e.g. early 8^th^ century BC, before the Hallstatt Plateau).

## Conclusion

We have presented here the first robust radiocarbon data and Bayesian model for Gezer covering the end of the LBA through Iron II period. The continuous sequence from the Tandy excavation has enabled us to anchor the absolute chronology of the site between the late 13^th^ and early 9^th^ centuries BC, addressing major changes throughout: four destruction layers, the fortification of Gezer during Iron I, the appearance of ‘Philistine’ material culture, and the contested date of Iron IIA monumental architecture. We have been able to help place developments at Gezer in regional perspective and check the chronological viability of multiple proposed historical correlations. Feasible from a strictly chronological point-of-view, is the connection of Merneptah with the end of Stratum 12B, the expansion of a highland polity with Stratum 8 and possibly Sheshonq I with the Stratum 8 destruction. Correlation of Siamun or Solomon with the end of Stratum 10A is unlikely and we can firmly rule out an association of the Stratum 7 destruction with Hazael’s campaign.

## Supporting information

S1 Appendix OxCal code for the Gezer Tandy excavation and for single-site models of neighboring sites used in Fig 11. (DOCX)

S1 Fig Alternative Bayesian model (Model C) using an agreement index approach to address outliers. (TIF)

S2 Fig Model B with a hypothetical radiocarbon offset of 19 ± 5 years applied. (TIF)

S1 Table Bayesian output, all models. Calendar estimates for strata and transitions (68.3% and 95.4% hpd). Shaded rows indicate where major cultural transitions occur. Estimates for strata were obtained using the ‘Date’ function in OxCal, while transitions are represented by phase boundaries.
(XLSX)

S2 Table Bayesian output, all models. Highest posterior densities (68.3% and 95.4%) for all dates.
(XLSX)
