# Supplementary material for: The chronology of Gezer from the end of the late bronze age to iron age II: A meeting point for radiocarbon, archaeology egyptology and the Bible
Source: PLoS One. 2023 Nov 15;18(11):e0293119. doi: 10.1371/journal.pone.0293119 (PMC10651010; doi:10.1371/journal.pone.0293119)
Supplement: S1 Appendix — (DOCX) [file pone.0293119.s001.docx]

**APPENDIX S1: OxCal Code**

**GEZER (outlier model)**

Options()

{

Resolution=1;

};

Plot()

{

Outlier_Model("General",T(5),U(0,3),"t");

Sequence("GEZER, Tandy excavation")

{

Boundary("Start 12")

{

color="green";

};

Phase("12B (XV)")

{

R_Date("GrM-13321 (94101)", 3090, 15)

{

Outlier("General",0.05);

};

R_Date("GrM-13317 (94052)", 3092, 15)

{

Outlier("General",0.05);

};

R_Date("GrM-13319 (94052)", 3005, 15)

{

Outlier("General",0.05);

};

R_Date("ETH-92839 (94052)", 2977, 22)

{

Outlier("General",0.05);

};

R_Date("SANU-60015 (94120)", 3005, 32)

{

Outlier("General",0.05);

};

R_Date("GrM-10986 (94032)", 2979, 17)

{

Outlier("General",0.05);

};

R_Date("Beta-436532 (82048)", 2970, 30)

{

Outlier("General",0.05);

};

R_Date("GrM-13322 (94108)", 2961, 15)

{

Outlier("General",0.05);

};

Date("Str. 12B estimate")

{

color="brown";

};

};

Boundary("Transition 12B to 12A")

{

color="green";

};

Phase("12A (XIV)")

{

Date("Str. 12A estimate")

{

color="brown";

};

};

Boundary("Transition 12A to 11")

{

color="green";

};

Phase("11 (XIII–XII)")

{

R_Date("OZ-V275 (92008)", 2990, 20)

{

Outlier("General",0.05);

};

R_Date("OZ-V270 (92030)", 2990, 20)

{

Outlier("General",0.05);

};

R_Date("OZ-V273 (92040)", 2955, 20)

{

Outlier("General",0.05);

};

R_Date("OZ-V272 (92040)", 2930, 25)

{

Outlier("General",0.05);

};

R_Date("GrM-10990 (92040)", 2873, 18)

{

Outlier("General",0.05);

};

Date("Str. 11 estimate")

{

color="brown";

};

};

Boundary("Transition 11 to 10B")

{

color="green";

};

Phase("10B (XI)")

{

R_Date("OZ-X997 (92010)", 2940, 25)

{

Outlier("General",0.05);

};

R_Combine("OZ-Z481/GrM-10988 (92010)")

{

Outlier("General",0.05);

R_Date("OZ-Z481 (92010)", 2865, 25);

R_Date("GrM-10988 (92010)", 2875, 18);

};

Date("Str. 10B estimate")

{

color="brown";

};

};

Boundary("Transition 10B to 10A")

{

color="green";

};

Phase("10A (X–IX)")

{

R_Combine("OZ-X996/OZ-Z480 (82040)")

{

Outlier("General",0.05);

R_Date("OZ-X996 (82040)", 2945, 25);

R_Date("OZ-Z480 (82040)", 2895, 25);

};

R_Date("Beta-436534 (82040)", 2910, 30)

{

Outlier("General",0.05);

};

R_Date("Beta-436535 (82026)", 2900, 30)

{

Outlier("General",0.05);

};

Date("Str. 10A estimate")

{

color="brown";

};

};

Boundary("Transition 10A to 9")

{

color="green";

};

Phase("9 (IX–VIII)")

{

R_Date("Beta-436533 (82023)", 2830, 30)

{

Outlier("General",0.05);

};

Date("Str. 9 estimate")

{

color="brown";

};

};

Boundary("End 9")

{

color="green";

};

Boundary("Start 8")

{

color="green";

};

Phase("8 (VIII)")

{

R_Date("Beta-436540 (81011)", 2980, 30)

{

Outlier("General",0.05);

};

R_Date("Beta-436538 (71042)", 2940, 30)

{

Outlier("General",0.05);

};

R_Date("Beta-436539 (71037)", 2800, 30)

{

Outlier("General",0.05);

};

R_Combine("OZ-X995/OZ-Z477 (71037)")

{

Outlier("General",0.05);

R_Date("OZ-X995 (71037)", 2815, 25);

R_Date("OZ-Z477 (71037)", 2785, 25);

};

Date("Str. 8 estimate")

{

color="brown";

};

};

Boundary("Transition 8 to 7")

{

color="green";

};

Phase("7 (VII)")

{

R_Date("OZ-V267 (91050)", 2860, 20)

{

Outlier("General",0.05);

};

R_Combine("OZ-X994/OZ-Z479 (81034)")

{

Outlier("General",0.05);

R_Date("OZ-X994 (81034)", 2845, 20);

R_Date("OZ-Z479 (81034)", 2840, 25);

};

R_Date("Beta-436537 (81034)", 2830, 30)

{

Outlier("General",0.05);

};

R_Combine("OZ-X993/OZ-Z478 (81002)")

{

Outlier("General",0.05);

R_Date("OZ-X993 (81002)", 2815, 20);

R_Date("OZ-Z478 (81002)", 2850, 25);

};

R_Date("GrM-13325 (81002)", 2762, 15)

{

Outlier("General",0.05);

};

R_Date("GrM-13324 (81002)", 2761, 15)

{

Outlier("General",0.05);

};

R_Date("Beta-436536 (81025)", 2730, 30)

{

Outlier("General",0.05);

};

Date("Str. 7 estimate")

{

color="brown";

};

};

Boundary("End 7")

{

color="green";

};

};

Axis(-1900,-700);

};

**Alternative GEZER model (elimination of lowest agreement indices)**

*Sequentially removed dates with the lowest agreement indices (Beta-436540, Beta-436538 then OZ-V267) until the overall model agreement index was >60%.*

Options()

{

Resolution=1;

};

Plot()

{

Sequence("GEZER, Tandy excavation")

{

Boundary("Start 12")

{

color="green";

};

Phase("12B (XV)")

{

R_Date("GrM-13321 (94101)", 3090, 15);

R_Date("GrM-13317 (94052)", 3092, 15);

R_Date("GrM-13319 (94052)", 3005, 15);

R_Date("ETH-92839 (94052)", 2977, 22);

R_Date("SANU-60015 (94120)", 3005, 32);

R_Date("GrM-10986 (94032)", 2979, 17);

R_Date("Beta-436532 (82048)", 2970, 30);

R_Date("GrM-13322 (94108)", 2961, 15);

Date("Str. 12B estimate")

{

color="brown";

};

};

Boundary("Transition 12B to 12A")

{

color="green";

};

Phase("12A (XIV)")

{

Date("Str. 12A estimate")

{

color="brown";

};

};

Boundary("Transition 12A to 11")

{

color="green";

};

Phase("11 (XIII–XII)")

{

R_Date("OZ-V275 (92008)", 2990, 20);

R_Date("OZ-V270 (92030)", 2990, 20);

R_Date("OZ-V273 (92040)", 2955, 20);

R_Date("OZ-V272 (92040)", 2930, 25);

R_Date("GrM-10990 (92040)", 2873, 18);

Date("Str. 11 estimate")

{

color="brown";

};

};

Boundary("Transition 11 to 10B")

{

color="green";

};

Phase("10B (XI)")

{

R_Date("OZ-X997 (92010)", 2940, 25);

R_Combine("OZ-Z481/GrM-10988 (92010)")

{

R_Date("OZ-Z481 (92010)", 2865, 25);

R_Date("GrM-10988 (92010)", 2875, 18);

};

Date("Str. 10B estimate")

{

color="brown";

};

};

Boundary("Transition 10B to 10A")

{

color="green";

};

Phase("10A (X–IX)")

{

R_Combine("OZ-X996/OZ-Z480 (82040)")

{

R_Date("OZ-X996 (82040)", 2945, 25);

R_Date("OZ-Z480 (82040)", 2895, 25);

};

R_Date("Beta-436534 (82040)", 2910, 30);

R_Date("Beta-436535 (82026)", 2900, 30);

Date("Str. 10A estimate")

{

color="brown";

};

};

Boundary("Transition 10A to 9")

{

color="green";

};

Phase("9 (IX–VIII)")

{

R_Date("Beta-436533 (82023)", 2830, 30);

Date("Str. 9 estimate")

{

color="brown";

};

};

Boundary("End 9")

{

color="green";

};

Boundary("Start 8")

{

color="green";

};

Phase("8 (VIII)")

{

//R_Date("Beta-436540 (81011)", 2980, 30);

//R_Date("Beta-436538 (71042)", 2940, 30);

R_Date("Beta-436539 (71037)", 2800, 30);

R_Combine("OZ-X995/OZ-Z477 (71037)")

{

R_Date("OZ-X995 (71037)", 2815, 25);

R_Date("OZ-Z477 (71037)", 2785, 25);

};

Date("Str. 8 estimate")

{

color="brown";

};

};

Boundary("Transition 8 to 7")

{

color="green";

};

Phase("7 (VII)")

{

//R_Date("OZ-V267 (91050)", 2860, 20);

R_Combine("OZ-X994/OZ-Z479 (81034)")

{

R_Date("OZ-X994 (81034)", 2845, 20);

R_Date("OZ-Z479 (81034)", 2840, 25);

};

R_Date("Beta-436537 (81034)", 2830, 30);

R_Combine("OZ-X993/OZ-Z478 (81002)")

{

R_Date("OZ-X993 (81002)", 2815, 20);

R_Date("OZ-Z478 (81002)", 2850, 25);

};

R_Date("GrM-13325 (81002)", 2762, 15);

R_Date("GrM-13324 (81002)", 2761, 15);

R_Date("Beta-436536 (81025)", 2730, 30);

Date("Str. 7 estimate")

{

color="brown";

};

};

Boundary("End 7")

{

color="green";

};

};

Axis(-1900,-700);

};

**MODELS USED FOR COMPARISON WITH NEIGHBORING SITES (FIGURE 11)**

**LACHISH**

*See summarised data and models in:*

Webster L. Synchronising the Chronologies of the Late Bronze Age Southern Levant and Egypt: A Radiocarbon Dating Perspective [PhD dissertation]. Vienna and Sydney: University of Vienna and Macquarie University; 2021.

**ASHKELON**

*Data:*

Asscher Y, Martin MAS, Master D, Boaretto E. A radiocarbon sequence for the Late Bronze to Iron Age transition at Ashkelon: timing early Philistine pottery. Bulletin of the American Schools of Oriental Research. 2021;386:77–93. doi: <https://doi.org/10.1086/714738>.

*Model:*

Options()

{

Resolution=1;

};

Plot()

{

Outlier_Model("General",T(5),U(0,3),"t");

Sequence("ASHKELON")

{

Boundary("Start 23B");

Phase("Phase 23B")

{

R_Date("RTD-6258", 3359, 45)

{

Outlier("General",0.05);

};

R_Date("RTD-6259", 3309, 45)

{

Outlier("General",0.05);

};

R_Date("RTD-6260", 3153, 49)

{

Outlier("General",0.05);

};

R_Date("RTD-6264", 3096, 24)

{

Outlier("General",0.05);

};

};

Boundary("23B to 22");

Phase("Phase 22")

{

};

Boundary("22 to 21");

Phase("Phase 21")

{

R_Date("RTD-8751", 2988, 38)

{

Outlier("General",0.05);

};

R_Date("RTD-8752", 2908, 27)

{

Outlier("General",0.05);

};

};

Boundary("21 to 20B");

Phase("Phase 20B")

{

R_Date("RTD-7574.1", 2939, 29)

{

Outlier("General",0.05);

};

R_Date("RTD-7574.2", 2927, 27)

{

Outlier("General",0.05);

};

};

Boundary("20B to 20A");

Phase("20A")

{

R_Date("RTD-7573.1", 2952, 27)

{

Outlier("General",0.05);

};

R_Date("RTD-7573.2", 2900, 39)

{

Outlier("General",0.05);

};

};

Boundary("20A to 19B");

Phase("Phase 19B")

{

R_Date("RTD-7572.1", 3027, 36)

{

Outlier("General",0.05);

};

R_Date("RTD-7572.2", 2956, 36)

{

Outlier("General",0.05);

};

};

Boundary("19B to 19A");

Phase("Phase 19A")

{

R_Date("RTD-7571.1", 2872, 36)

{

Outlier("General",0.05);

};

R_Date("RTD-7571.2", 2952, 31)

{

Outlier("General",0.05);

};

};

Boundary("19A to 18B");

Phase("Phase 18B")

{

R_Date("RTD-7570.1", 2946, 32)

{

Outlier("General",0.05);

};

R_Date("RTD-7570.2", 2825, 28)

{

Outlier("General",0.05);

};

};

Boundary("18B to 18A");

Phase("Phase 18A")

{

R_Date("RTD-7569.1", 2964, 26)

{

Outlier("General",0.05);

};

R_Date("RTD-7569.2", 2852, 44)

{

Outlier("General",0.05);

};

};

Boundary("18A to 17B");

Phase("Phase 17B")

{

R_Date("RTD-7568.1", 2925, 31)

{

Outlier("General",0.05);

};

R_Date("RTD-7568.2", 2898, 26)

{

Outlier("General",0.05);

};

};

Boundary("End 17B");

};

};

**TELL ES-SAFI**

*Data:*

Sharon I, Gilboa A, Jull AJT, Boaretto E. Report on the first stage of the Iron Age dating project in Israel: supporting a low chronology. Radiocarbon. 2007;49(1):1–46. doi: <https://doi.org/10.1017/S0033822200041886>.

Asscher Y, Cabanes D, Hitchcock LA, Maeir AM, Weiner S, Boaretto E. Radiocarbon dating shows an early appearance of Philistine material culture in Tell es-Safi/Gath, Philistia. Radiocarbon. 2015;57(5):825–50. doi: <https://doi.org/10.2458/azu_rc.57.18391>.

*Model:*

Options()

{

Resolution=1;

};

Plot()

{

Outlier_Model("General",T(5),U(0,3),"t");

Sequence("TELL ES-SAFI, Area A")

{

Boundary("Start A7");

Phase("A7")

{

R_Date("RTD-7098", 3023, 42)

{

Outlier("General", 0.05);

};

R_Date("RTD-6711", 3029, 45)

{

Outlier("General", 0.05);

};

};

Boundary("End A7");

Interval("Interval A7 to A6");

Boundary("Start A6");

Sequence("A6")

{

R_Date("RTD-7095", 2968, 30);

Phase("A6")

{

R_Date("RTK-6579", 2925, 55)

{

Outlier("General", 0.05);

};

R_Date("RTK-6580", 2930, 55)

{

Outlier("General", 0.05);

};

R_Date("RTK-6581", 2950, 55)

{

Outlier("General", 0.05);

};

};

R_Combine("RTD-6983")

{

Outlier("General", 0.05);

R_Date("RTD-6983.1", 2945, 32);

R_Date("RTD-6983.2", 2860, 45);

};

};

Boundary("A6 to A5");

Phase("A5")

{

Interval("Interval A6 to A4");

};

Boundary("A5 to A4");

Sequence("A4")

{

R_Date("RTD-7200", 2880, 30)

{

Outlier("General", 0.05);

};

R_Date("RTK-5940", 2850, 55)

{

Outlier("General", 0.05);

};

};

Boundary("End A4");

Boundary("Start A3");

Phase("A3")

{

R_Date("RT-4409.3", 2630, 45)

{

Outlier("General",0.05);

};

R_Date("RT-4409.4", 2693, 60)

{

Outlier("General",0.05);

};

R_Date("RT-4409.5", 2679, 55)

{

Outlier("General",0.05);

};

R_Date("RT-4410.3", 2748, 60)

{

Outlier("General",0.05);

};

R_Date("RT-4410.4", 2671, 45)

{

Outlier("General",0.05);

};

R_Date("RT-4410.5", 2712, 45)

{

Outlier("General",0.05);

};

R_Date("GrA-25536", 2700, 42)

{

Outlier("General",0.05);

};

R_Date("GrA-25711", 2733, 38)

{

Outlier("General",0.05);

};

R_Date("GrA-25770", 2780, 44)

{

Outlier("General",0.05);

};

};

Boundary("End A3");

};

Axis(-2000,-500);

};

**TEL MIQNE (EKRON)**

*Data:*

Sharon I, Gilboa A, Jull AJT, Boaretto E. Report on the first stage of the Iron Age dating project in Israel: supporting a low chronology. Radiocarbon. 2007;49(1):1–46. doi: <https://doi.org/10.1017/S0033822200041886>.

Asscher Y, Boaretto E. Absolute time ranges in the plateau of the Late Bronze to Iron Age transition and the appearance of Bichrome pottery in Canaan, Southern Levant. Radiocarbon. 2018;61(1):13–37. doi: <https://doi.org/10.1017/RDC.2018.58>.

*Model:*

Options()

{

Resolution=1;

};

Plot()

{

Outlier_Model("General",T(5),U(0,3),"t");

Sequence("EKRON")

{

Boundary("Start VIII");

Phase("VIII occupation (Field I)")

{

};

Boundary();

Phase("VIII destruction (Field I)")

{

R_Date("RT-4430", 2980, 40)

{

Outlier("General",0.05);

};

R_Date("RT-4431", 2995, 40)

{

Outlier("General",0.05);

};

};

Boundary("End VIII");

Boundary("Start VIIB");

Phase("VIIB (Field IV)")

{

R_Date("RTT-4286.3", 2950, 55)

{

Outlier("General",0.05);

};

R_Date("RTT-4286.4", 2900, 40)

{

Outlier("General",0.05);

};

R_Date("RTT-4286.5", 2870, 60)

{

Outlier("General",0.05);

};

};

Boundary("Transition VIIB to VIIA");

Phase("VIIA")

{

};

Boundary("Transition VIIA to VIB");

Phase("VIB (Field IV)")

{

R_Date("RTT-4283.3", 2915, 45)

{

Outlier("General",0.05);

};

R_Date("RTT-4283.4", 2960, 45)

{

Outlier("General",0.05);

};

R_Date("RTT-4283.5", 2880, 45)

{

Outlier("General",0.05);

};

};

Boundary("Transition VIB to VIA");

Phase("VIA")

{

};

Boundary("Transition VIA to VB");

Phase("VB (Field IV)")

{

R_Date("RTT-4282.3", 2894, 53)

{

Outlier("General",0.05);

};

R_Date("RTT-4282.4", 2837, 42)

{

Outlier("General",0.05);

};

R_Date("RTT-4282.5", 2899, 47)

{

Outlier("General",0.05);

};

R_Date("RTT-4282a", 2872, 36)

{

Outlier("General",0.05);

};

R_Date("RTT-4282aa", 2895, 38)

{

Outlier("General",0.05);

};

R_Date("RTT-4284.3", 2835, 45)

{

Outlier("General",0.05);

};

R_Date("RTT-4284.4", 2830, 45)

{

Outlier("General",0.05);

};

};

Boundary("End VB");

};

Axis(-1700,-700);

};

**BETH SHEMESH**

*Data:*

Boaretto E, Sharon I, Gilboa A. Radiocarbon dating of the Iron Age stratigraphic sequence. In: Bunimovitz S, Lederman Z, editors. Tel Beth-Shemesh: A Border Community in Judah: Renewed Excavations 1990–2000 The Iron Age. Winona Lake: Eisenbrauns; 2016. p. 680–7.

Piasetzky E. Radiocarbon dating at Tel Beth-Shemesh: a second look. In: Bunimovitz S, Lederman Z, editors. Tel Beth-Shemesh: A Border Community in Judah: Renewed Excavations 1990–2000 The Iron Age. Winona Lake: Eisenbrauns; 2016. p. 688–94.

*Model:*

Options()

{

Resolution=1;

};

Plot()

{

Outlier_Model("General",T(5),U(0,3),"t");

Sequence("BETH SHEMESH")

{

Boundary("Start 6");

Phase("6")

{

R_Combine("RTT-3934")

{

Outlier("General",0.05);

R_Date("RTT-3934.3", 2830, 50);

R_Date("RTT-3934.4", 2925, 50);

R_Date("RTT-3934.5", 2810, 50);

};

};

Boundary("Transition 6 to 5");

Phase("5")

{

R_Date("3935.3", 2830, 53)

{

Outlier("General",0.05);

};

R_Date("3935.4", 2750, 55)

{

Outlier("General",0.05);

};

R_Date("3935.5", 2770, 65)

{

Outlier("General",0.05);

};

R_Date("3936.3", 2810, 50)

{

Outlier("General",0.05);

};

R_Date("3936.4", 2850, 55)

{

Outlier("General",0.05);

};

R_Date("3936.5", 2855, 65)

{

Outlier("General",0.05);

};

};

Boundary("Transition 5 to 4");

Phase("4")

{

R_Date("OxA 26129", 2935, 26)

{

Outlier("General",0.05);

};

R_Date("OxA 26130", 2817, 25)

{

Outlier("General",0.05);

};

R_Date("OxA 26131", 2835, 25)

{

Outlier("General",0.05);

};

};

Boundary("End 4");

Boundary("Start 3");

Phase("3 early")

{

R_Date("ETH-30461", 2645, 45)

{

Outlier("General",0.05);

};

R_Date("ETH-30462", 2660, 45)

{

Outlier("General",0.05);

};

R_Date("ETH-30463", 2825, 45)

{

Outlier("General",0.05);

};

};

Boundary();

Phase("3 destruction")

{

R_Date("3937.1", 2500, 35)

{

Outlier("General",0.05);

};

R_Date("3937.3", 2524, 36)

{

Outlier("General",0.05);

};

R_Date("3937.4", 2427, 35)

{

Outlier("General",0.05);

};

R_Date("3937.5", 2478, 34)

{

Outlier("General",0.05);

};

R_Date("3938.3", 2390, 65)

{

Outlier("General",0.05);

};

R_Date("3938.4", 2425, 40)

{

Outlier("General",0.05);

};

R_Date("3938.5", 2505, 40)

{

Outlier("General",0.05);

};

};

Boundary("End 3");

};

Axis(-1800,-400);

};

**KHIRBET AL-RAI**

*Data:*

Garfinkel Y, Hasel MG, Klingbeil MG, Kang HG, Choi G, Chang S-Y, et al. Lachish fortifications and state formation in the biblical kingdom of Judah in light of radiometric datings. Radiocarbon. 2019;61(3):695–712. doi: <https://doi.org/10.1017/RDC.2019.5>.

*Model:*

Options()

{

Resolution=1;

};

Plot()

{

Outlier_Model("General",T(5),U(0,3),"t");

Sequence("KHIRBET AL-RA'I ")

{

Boundary("Start VIII");

Phase("Level VIII")

{

R_Date("OxA-33476 ", 2969, 31)

{

Outlier("General", 0.05);

};

R_Date("OxA-33475 ", 2929, 29)

{

Outlier("General", 0.05);

};

R_Date("OxA-33477", 2899, 29)

{

Outlier("General", 0.05);

};

R_Date("OxA-33468", 2829, 29)

{

Outlier("General", 0.05);

};

R_Date("OxA-33469", 2825, 30)

{

Outlier("General", 0.05);

};

};

Boundary("End VIII");

Boundary("Start VII");

Phase("Level VII")

{

R_Date("OxA-34501", 2922, 30)

{

Outlier("General", 0.05);

};

R_Date("OxA-34969", 2878, 30)

{

Outlier("General", 0.05);

};

R_Date("OxA-34970", 2842, 30)

{

Outlier("General", 0.05);

};

};

Boundary("End VII");

};

Axis(-1600,-800);

};

**QEIYAFA**

*Data:*

Garfinkel Y, Streit K, Ganor S, Hasel MG. State formation in Judah: biblical tradition, modern historical theories, and radiometric dates at Khirbet Qeiyafa. Radiocarbon. 2012;54(3–4):359–369. doi: <https://doi.org/10.1017/S0033822200047147>.

Garfinkel Y, Streit K, Ganor S, Reimer PJ. King David's city at Khirbet Qeiyafa: results of the second radiocarbon dating project. Radiocarbon. 2015;57(5):881–90. doi: https://doi.org/10.2458/azu_rc.57.17961.

*Model:*

Options()

{

Resolution=1;

};

Plot()

{

Outlier_Model("General",T(5),U(0,3),"t");

Sequence("QEIYAFA")

{

Boundary("Start");

Phase()

{

R_Date("OxA-19127",2910,26)

{

Outlier("General",0.05);

};

R_Date("OxA-19589",2883,29)

{

Outlier("General",0.05);

};

R_Date("OxA-22044",2858,33)

{

Outlier("General",0.05);

};

R_Date("OxA-23505",2852,26)

{

Outlier("General",0.05);

};

R_Date("OxA-19425",2851,31)

{

Outlier("General",0.05);

};

R_Date("OxA-23506",2843,26)

{

Outlier("General",0.05);

};

R_Date("OxA-19426",2837,29)

{

Outlier("General",0.05);

};

R_Date("OxA-22045",2830,30)

{

Outlier("General",0.05);

};

R_Date("OxA-23504",2827,27)

{

Outlier("General",0.05);

};

R_Date("OxA-19588",2799,31)

{

Outlier("General",0.05);

};

Label("Jar C11747");

R_Combine()

{

Outlier("General",0.05);

R_Date("UBA-22138", 2840, 31);

R_Date("OxA-27783", 2825, 26);

};

R_Combine()

{

Outlier("General",0.05);

R_Date("UBA-22139", 2790, 29);

R_Date("OxA-27612", 2838, 27);

};

R_Combine()

{

Outlier("General",0.05);

R_Date("UBA-22140", 2895, 28);

R_Date("OxA-27747", 2823, 27);

};

R_Combine()

{

Outlier("General",0.05);

// R_Date("UBA-22141a", 2988, 46);

R_Date("UBA-22141b", 2806, 32);

R_Date("OxA-27613", 2884, 28);

};

R_Date("UBA-22142", 2868, 37)

{

Outlier("General",0.05);

};

R_Date("UBA-22143", 2847, 40)

{

Outlier("General",0.05);

};

R_Date("UBA-22144", 2896, 33)

{

Outlier("General",0.05);

};

R_Date("UBA-22145", 2903, 29)

{

Outlier("General",0.05);

};

R_Date("UBA-22146a", 2757, 31)

{

Outlier("General",0.05);

};

R_Date("UBA-22146b", 2871, 29)

{

Outlier("General",0.05);

};

R_Date("UBA-22147", 2776, 38)

{

Outlier("General",0.05);

};

R_Date("OxA-27748", 2790, 27)

{

Outlier("General",0.05);

};

};

Boundary("End");

};

};

**TEL ZAYIT**

*Data:*

Sharon I, Gilboa A, Jull AJT, Boaretto E. Report on the first stage of the Iron Age dating project in Israel: supporting a low chronology. Radiocarbon. 2007;49(1):1–46. doi: <https://doi.org/10.1017/S0033822200041886>.

Tappy, RE, McCarter, PK, Lundberg, MJ, Zuckerman, B. An abecedary of the mid-tenth century B.C.E. from the Judaean Shephelah. Bulletin of the American Schools of Oriental Research. 2006;344:5–46. doi: https://doi.org/10.1086/BASOR25066976.

*Model:*

Options()

{

Resolution=1;

};

Plot()

{

Outlier_Model("General",T(5),U(0,3),"t");

Outlier_Model("Charcoal_p",Prior("Charcoal_p.prior"),U(0,3),"t");

Sequence("TEL ZAYIT")

{

Boundary("Start Level I");

Phase("Level I")

{

R_Date("Gr#1 (2503)", 2750, 20)

{

Outlier("General",0.05);

};

R_Date("Gr#2 (1476)", 2730, 40)

{

Outlier("General",0.05);

};

R_Date("RT-4279.4 (1476)", 2681, 35)

{

Outlier("Charcoal_p",1);

};

R_Date("RT-4279.5 (1476)", 2683, 35)

{

Outlier("Charcoal_p",1);

};

R_Date("RT-4280.3 (1476)", 2615, 40)

{

Outlier("Charcoal_p",1);

};

R_Date("RT-4280.4 (1476)", 2653, 40)

{

Outlier("Charcoal_p",1);

};

R_Date("RT-4275-1.3 (1477)", 2640, 40)

{

Outlier("General",0.05);

};

R_Date("RT-4275-1.4 (1477)", 2646, 45)

{

Outlier("General",0.05);

};

R_Date("RT-4275-1.5 (1477)", 2745, 55)

{

Outlier("General",0.05);

};

R_Date("RT-4275-2.3 (1477)", 2616, 40)

{

Outlier("General",0.05);

};

R_Date("RT-4275-3.3 (1477)", 2573, 40)

{

Outlier("Charcoal_p",1);

};

R_Date("RT-4275-3.4 (1477)", 2605, 40)

{

Outlier("Charcoal_p",1);

};

R_Date("RT-4275-3.5 (1477)", 2600, 40)

{

Outlier("Charcoal_p",1);

};

R_Date("RT-4278.3 (1477)", 1400, 35)

{

Outlier();

};

R_Date("RT-4278.4 (1477)", 1390, 35)

{

Outlier();

};

R_Date("RT-4278.5 (1477)", 1455, 35)

{

Outlier();

};

};

Boundary("End Level I");

};

Axis(-1900,-500);

};
