# Supplementary figures and images for: The chronology of Gezer from the end of the late bronze age to iron age II: A meeting point for radiocarbon, archaeology egyptology and the Bible
Source: PLoS One. 2023 Nov 15;18(11):e0293119. doi: 10.1371/journal.pone.0293119 (PMC10651010; doi:10.1371/journal.pone.0293119)

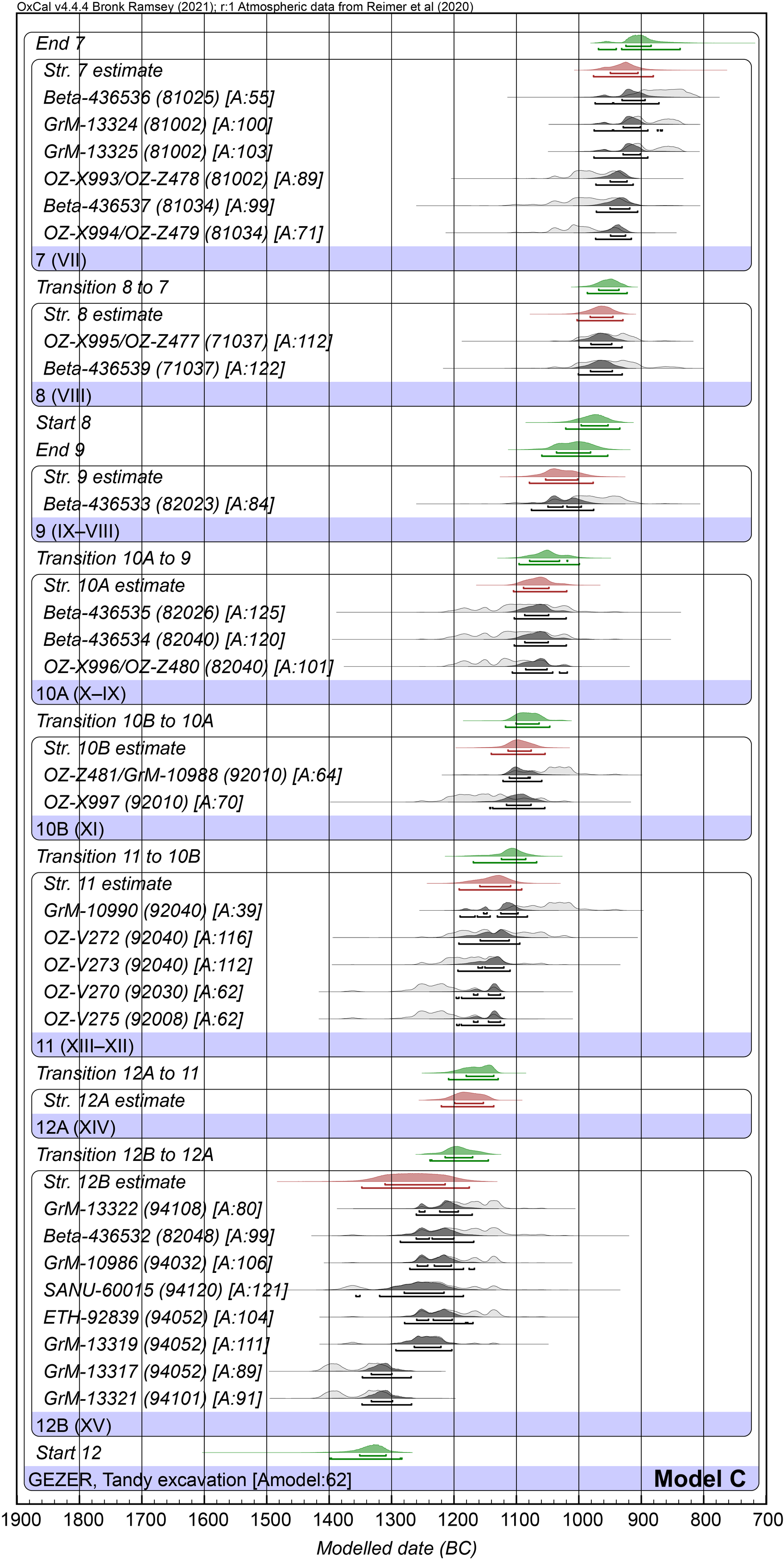

Supplement: S1 Fig — (TIF) [file pone.0293119.s002.tif]

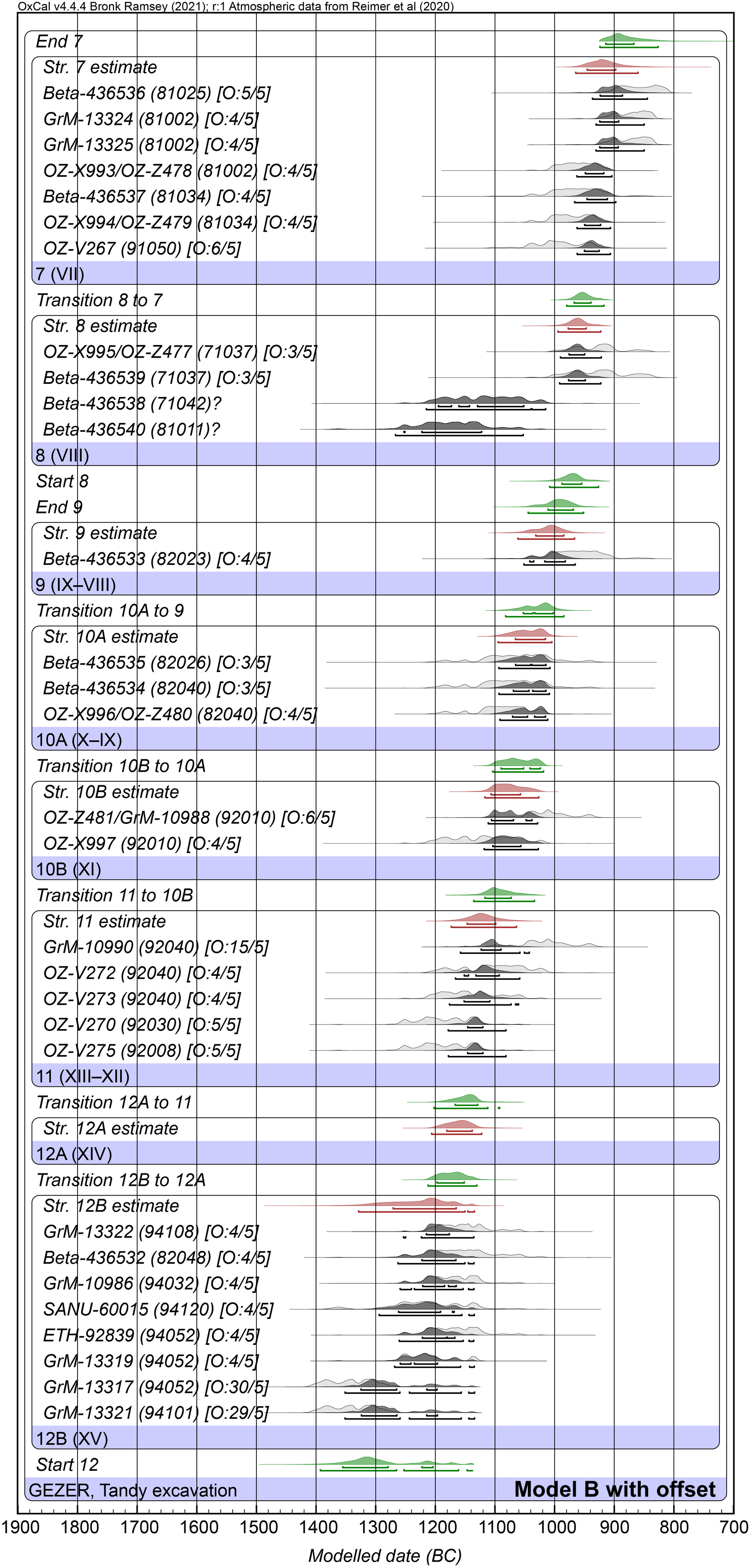

Supplement: S2 Fig — (TIF) [file pone.0293119.s003.tif]
